# Bioenergetics of aerobic and anaerobic growth of *Shewanella putrefaciens* CN32

**DOI:** 10.3389/fmicb.2023.1234598

**Published:** 2023-08-02

**Authors:** Addien C. Wray, Drew Gorman-Lewis

**Affiliations:** Earth and Space Sciences, University of Washington, Seattle, WA, United States

**Keywords:** *Shewanella putrefaciens* CN32, dissimilatory iron-reducing bacterium, bioenergetics, Gibbs energy consumption, growth enthalpy

## Abstract

*Shewanella putrefaciens* is a model dissimilatory iron-reducing bacterium that can use Fe(III) and O_2_ as terminal electron acceptors. Consequently, it has the ability to influence both aerobic and anaerobic groundwater systems, making it an ideal microorganism for improving our understanding of facultative anaerobes with iron-based metabolism. In this work, we examine the bioenergetics of O_2_ and Fe(III) reduction coupled to lactate oxidation in *Shewanella putrefaciens* CN32. Bioenergetics were measured directly via isothermal calorimetry and by changes to the chemically defined growth medium. We performed these measurements from 25 to 36°C. Modeling metabolism with macrochemical equations allowed us to define a theoretical growth stoichiometry for the catabolic reaction of 1.00 O_2_:lactate and 1.33 Fe(III):lactate that was consistent with the observed ratios of O_2_:lactate (1.20 ± 0.23) and Fe(III):lactate (1.46 ± 0.15) consumption. Aerobic growth showed minimal variation with temperature and minimal variation in thermodynamic potentials of incubation. Fe(III)-based growth showed a strong temperature dependence. The Gibbs energy and enthalpy of incubation was minimized at ≥30°C. Energy partitioning modeling of Fe(III)-based calorimetric incubation data predicted that energy consumption for non-growth associate maintenance increases substantially above 30°C. This prediction agrees with the data at 33 and 35°C. These results suggest that the effects of temperature on *Shewanella putrefaciens* CN32 are metabolism dependent. Gibbs energy of incubation above 30°C was 3–5 times more exergonic with Fe(III)-based growth than with aerobic growth. We compared data gathered in this study with predictions of microbial growth based on standard-state conditions and based on the thermodynamic efficiency of microbial growth. Quantifying the growth requirements of *Shewanella putrefaciens* CN32 has advanced our understanding of the thermodynamic constraints of this dissimilatory iron-reducing bacterium.

## 1. Introduction

Microbial metabolism play a key role in the establishment of the geochemistry of groundwater systems (Flynn et al., 2013; Maamar et al., 2015; Mansuy, 2018). To best predict potential interactions between microbes and their geochemical environment in a given system, it is necessary to consider the bioenergetics involved (Heijnen and Kleerebezem, 2010; Smeaton and Van Cappellen, 2018; Cook et al., 2021; Hart and Gorman-Lewis, 2021; Paquete et al., 2021). Microbial bioenergetics are mainly based on redox reactions performed during the catabolic cycle (Heijnen and Kleerebezem, 2010). This allows us to describe geochemical systems in terms of the potential available chemical energy, based on available electron donors and acceptors (Hoehler, 2004; Ray et al., 2021). For example, in a system with available organic carbon, the potential free energy for heterotrophic growth is maximized when the oxidation of that organic carbon is coupled to O_2_ reduction (von Stockar and Liu, 1999; Heijnen and Kleerebezem, 2010). In the absence of sufficient O_2_, other potential electron acceptors, such as Fe(III), become metabolically relevant. Similarly, in anoxic and dysoxic systems, a variety of redox couples are important, where the maximum available energy depends on the available redox couples (Amenabar et al., 2017). In addition to O_2_ and other metabolically-available gases (e.g., CO_2_), colloidal and dissolved minerals are metabolically relevant when considering the entire subsurface environment (O'Loughlin et al., 2019). As a result, highly diverse microbial communities and taxa that can metabolize a variety of available energy sources play a major role in establishing subsurface redox conditions (Borch et al., 2010; Sang et al., 2018).

The facultative anaerobic bacterium *Shewanella putrefaciens* is widely considered a model dissimilatory metal reducing bacterium (DMRB), as it can obtain energy for growth on a variety of metals (Lovley, 1993; Liu et al., 2002; Brooks et al., 2003; Yang et al., 2014). *S. putrefaciens* is unique among most DMRBs because it is also able to grow aerobically, making it relevant to both oxic and anaerobic systems (Moser and Nealson, 1996). However, to date, little research has compared its aerobic and anaerobic growth strategies. This is an important gap in the literature, as the metabolic plasticity exhibited by *S. putrefaciens* is relevant to the impact it may have on metals in the subsurface. Specifically, it allows the organism to maintain its growth even if metal concentrations fluctuate, potentially resulting in varied redox conditions, which would affect other microbial metabolism and thus the geochemical state of the system as a whole.

The growth of a variety of *S. putrefaciens* strains and metabolism have been partially investigated stoichiometrically. However, little work has sought to verify theoretical catabolic stoichiometry with more detailed measurements of changes to growth media. For example, Kostka et al. (1995) first identified strain MR-1 as a catalyst for Mn(III) reduction, but only measured the relative changes in electron donors and acceptors. A similar analysis was performed by Philips et al. (2018) for Fe(0) oxidation by strain 4t3-1-2LB. The stoichiometry of Fe(II) mineral products from Fe(III) reduction-based enzymatic activity by strain CN32 has also been investigated (Kukkadapu et al., 2005). Growth-based Fe(III) reduction in strains W3-18-1 and CN32 was also investigated by Salas et al. (2009) and O'Loughlin et al. (2019), respectively, where the stoichiometric ratios of the electron donors, Fe(II), and in the case of Salas et al. (2009), carbon sources, were measured. To the best knowledge of the authors, measurements pertaining to the stoichiometry of aerobic growth by *S. putrefaciens* have not been made, nor has the stoichiometry of aerobic or anaerobic growth been considered explicitly taking into account the anabolic reaction. To fully understand the impact that a microbe may have on its environment, observations of growth stoichiometries are necessary. The lack of these data and the dependence on theoretical catabolic reactions is a crucial gap in the literature and must be addressed to accurately assess the impact *S. putrefaciens* may have on *in situ* geochemical conditions throughout the subsurface.

Accurate descriptions of growth stoichiometry are also necessary to characterize growth in terms of chemical energy consumption. Microbial cells consume nutrients in growth media to harvest energy with catabolic reactions. This harvested energy is used to drive the anabolic (biosynthetic) reactions and other reactions associated with maintaining cellular viability (Heijnen and Kleerebezem, 2010; Calabrese et al., 2021). Coupling these reactions together we can quantitatively describe the overall growth process. At constant temperature and pressure, chemical energy consumption is conveniently described by changes in Gibbs energy. We can use simplified chemical equations to represent catabolism and anabolism (Heijnen and Kleerebezem, 2010; Calabrese et al., 2021; Hunt et al., 2022). It is possible to quantify the Gibbs energy consumed during growth using chemical changes in the growth media combined with simplified chemical equations representing growth (Smith and Shock, 2007; Hunt et al., 2022). This Gibbs energy consumption characterizes geochemical impacts on the environment and provides a means to assess the potential for growth based on geochemical conditions.

A more complete characterization of microbial growth includes calorimetric measurements of growth in addition to calculations of Gibbs energy consumption. Heat, energy that is transferred from one system to its surroundings because of a temperature difference, is a direct result of Gibbs energy consumption during microbial growth, and calorimetry is an ideal means to measure that energy transfer (Maskow and Paufler, 2015). Calorimetric measurements are a direct measurement of real-time metabolic activity (Gnaiger, 1990; Gustafsson, 1991; Wadso, 1997). Therefore, calorimetric measurements directly correspond to growth curves based on cell counts, substrate consumption, and metabolite production, allowing one to understand rates of energy transfer during microbial growth (e.g., Traore et al., 1981; Russell, 1986; Braissant et al., 2010a,b; Hart and Gorman-Lewis, 2021). At constant temperature and pressure, calorimetric measurements correspond to enthalpy (Δ*H*). Equation (1) describes the relationship between Gibbs energy consumption, enthalpy, and entropy (Δ*S*), where T is absolute temperature. A combined analysis of Δ*G* and Δ*H* for microbial growth has only rarely been applied (e.g., von Stockar et al., 1993b, 2006; Schill et al., 1999; Hart and Gorman-Lewis, 2021; Hunt et al., 2022), but yields a more nuanced description of the overall energetics involved in growth. Recently, Hunt et al. (2022) demonstrated how calorimetric growth measurements could be used to determine energy partitioning into growth-associated costs and non-growth-associated maintenance as a function of temperature stress. This approach has not been applied to any *S. putrefaciens* strains. Geochemical conditions are ultimately impacted by the metabolic performance of microbes, and that is heavily influenced by energy partitioning between growth-associated costs and non-growth-associated maintenance. Characterizing *S. putrefaciens* CN32 growth with calorimetry provides the means to understand those chemical changes in terms of energetic costs under temperature stress.


(1) $$\begin{array}{l} \Delta H=\Delta G+T\Delta S \end{array}$$

A complete understanding of *S. putrefaciens* CN32 biology and its impacts on subsurface geochemistry should include a stoichiometric and thermodynamic description of its aerobic and anaerobic growth. In this study, we examine lactate oxidation by *S. putrefaciens* strain CN32 coupled to O_2_ and Fe(III) reduction via isothermal calorimetry and through measured changes in a chemically defined growth medium. Furthermore, by repeating these measurements over a range of temperatures (25–36°C), we were able to infer how *S. putrefaciens* allocates its energy under a variety of conditions.

## 2. Materials and methods

### 2.1. Culture preparation

*S. putrefaciens* strain CN32 (CN32) was maintained aerobically and anaerobically in a defined growth medium based on Myers and Nealson (1988) and Belli et al. (2015) at 30°C (additional details in Supplementary material). Aerobic cultures were grown with approximately 10 mM sodium lactate in 15 ml borosilicate glass tubes with aerobic cap closures on an orbital shaker. Anaerobic cultures were grown with approximately 15 mM sodium lactate and 30 mM ferric citrate, in borosilicate glass tubes sealed with butyl rubber septum-type stoppers and aluminum seal rings. The anaerobic chamber was maintained with an N_2_:H_2_ atmosphere (98:2). Cultures were maintained with an initial cell density of 8 × 10^5^ (±2 × 10^5^) cells per mL, and were grown to late exponential phase. Cell count slides were prepared using polycarbonate 0.2 μm filter membranes with SYBR Green I dye following the procedure of Lunau et al. (2005) and with appropriate filters on a Zeiss Axiostar Plus microscope.

### 2.2. Calorimetric analysis

Calorimetric measurements were made on a TAM III nanocalorimeter measuring heat flux between a reaction and the reference vessel as a function of time (Johansson and Wadsö, 1999; Goldberg and Wadsö, 2001). The calorimetric response was calibrated by electrical heating and verified by measuring the protonation of TRIS at 25°C (Grenthe et al., 1970). Three milliliters of experimental cultures were grown in 4.22 mL glass vials, sealed with butyl rubber septa and aluminum sealing rings without agitation. Experiments were performed from 25 to 36°C with 2 replicates at each temperature.

To maintain media compositions similar to the maintenance cultures, aerobic and anaerobic cultures were prepared with the same base medium with an initial cell density of 5 × 10^5^ (±1 × 10^5^) cells per mL. Initial sodium lactate concentrations were kept the same as in the maintenance cultures, and the electron acceptor was limited for aerobic and anaerobic growth. Aerobic cultures were sealed under atmospheric conditions, yielding an initial headspace of approximately 10 μmol O_2_. For the anaerobic cultures, the initial concentration of ferric citrate was approximately 7 mM, and the vials were sealed in the anaerobic chamber.

The total heat evolved during growth was measured by monitoring the heat flux in the calorimeter throughout the growth period and integrating under the resulting curve (see Supplementary material). Calculation of microbial growth rate was based on total heat evolution using an exponential model. The duration of lag phase was determined using miLag (Opalek et al., 2022).

### 2.3. Overall growth reaction modeling

The systems investigated in this work relate to the growth of CN32 under controlled conditions in reaction vials. From this point on, we refer to changes in the reaction vials as a result of microbial growth as “incubation” to recognize that abiotic or biotically influenced abiotic reactions will also occur. Simplified macrochemical equations were used to represent the growth of one carbon-mole (C-mol) of generic biomass, according to the formula CH_1.8_O_0.5_N_0.2_ (Heijnen and Kleerebezem, 2010; Calabrese et al., 2021; Hunt et al., 2022). Microbial biomass formulas are known to have very similar elemental compositions (Popovic, 2019), and a 10% error in the biomass formula weight was propagated through calculations to take into account variations. The anabolic and catabolic components of the growth reaction are represented according to Equations (2) and (3). The coefficients for the anabolic and catabolic reactions were determined by solving a series of linear equations to satisfy balances of mass and charge.


(2) $$\begin{array}{c} Y^{an}=a\cdot e^{-}\text{donor}+b\cdot \text{N source}+c\cdot H^{+}+d\cdot H_{2}O\\ +e\cdot CO_{2}+f\cdot \text{oxidized}e^{-}\text{donor}+CH_{1.8}O_{0.5}N_{0.2} \end{array}$$

(3) $$\begin{array}{l} Y^{cat}=g\cdot e^{-}\text{donor}\;+\;h\cdot e^{-}\text{acceptor}\;+\;i\cdot \text{oxidized}e^{-}\text{donor}\\ +\;j\cdot \text{reduced}e^{-}\text{acceptor} \end{array}$$

To produce sufficient energy to drive anabolism, the catabolic reaction must be performed multiple times. This relationship defines the catabolic multiplicative factor necessary to produce one C-mol of biomass, *f*_*cat*_, as described in Equation (4)


(4) $$\begin{array}{l} f_{cat}=\frac{-1}{Y_{X/D}}+Y_{D}^{an} \end{array}$$

where *Y*_*X*/*D*_ is the C-mol of biomass produced per mol of the *e*^−^ donor consumed and $Y_{D}^{an}$ is the anabolic coefficient of the *e*^−^ donor.

From Equations (2)–(4), we can calculate the coefficients for the overall growth reaction (OGR) with Equation (5):


(5) $$\begin{array}{l} Y^{OGR}=Y^{an}+f_{cat}\cdot Y^{cat} \end{array}$$

### 2.4. Gibbs energy of incubation

The Gibbs energy change during incubation was calculated for each experiment before and after growth on the basis of the activities of all the chemical species in the overall growth reaction. Measurements of pH and O_2_ were made with an Orion 8103BN Ross semi-micro combination pH electrode and a Clark-type OX-NP-006340 oxygen microelectrode from Unisense, respectively. Fe(III) was measured with the ferrozine method (Viollier et al., 2000) under anaerobic conditions. Lactate was measured with a spectrophotometric assay according to Borshchevskaya et al. (2016). Biomass produced during incubation was determined by difference between final cell counts and initial cell counts. Standard states for this work at all temperatures and pressures are the unit activity of the pure solvent, the unit activity of aqueous species in a hypothetical 1 molal solution referenced to infinite dilution, the unit activity of pure minerals or other crystalline solids, and the unit fugacity of a pure gas at 1 bar. The standard state Gibbs energies of formation ($\Delta G_{f}^{{}^{\circ}}$) for the chemical species in the growth reaction were calculated at experiment temperature with the revised Helgeson-Kirkham-Flowers equations of state (HKF) (Helgeson et al., 1978, 1981; Tanger and Helgeson, 1988) using SUPCRT92 (Shock et al., 1989; Johnson et al., 1992). For biomass, $\Delta G_{f}^{{}^{\circ}}=-67$ kJ/mol was used for all growth experiments (Heijnen and van Dijken, 1992). The standard-state Gibbs energies of reactions ($\Delta G_{r}^{{}^{\circ}}$) of the overall growth reaction determined for each experiment were calculated using Equation (6), where $\Delta G_{f,products}^{{}^{\circ}}$ and $G_{f,reactants}^{{}^{\circ}}$ are the standard-state Gibbs energies of formation for the products and reactants of the growth reaction, respectively, and $Y_{i}^{ogr}$ is the stoichiometric coefficient of the *i*th chemical species in the overall growth reaction.


(6) $$\begin{array}{l} \Delta G_{r}^{{}^{\circ}}=\sum \left(\Delta \overset{{}^{\circ}}{\underset{f,products}{G}}\times Y_{i}^{ogr}\right)-\sum \left(\Delta \overset{{}^{\circ}}{\underset{f,reactants}{G}}\times Y_{i}^{ogr}\right) \end{array}$$

The change in Gibbs energy of a given chemical reaction (Δ*G*_*r*_) represents the Gibbs energy potentially available for microbial growth. This is defined by Equation (8), where $\Delta G_{r}^{{}^{\circ}}$ is modified with the concentration-dependent reaction quotient (*Q*), the universal gas constant (*R*), and absolute temperature (*T*).


(7) $$\begin{array}{l} \Delta G=\Delta G_{r}^{{}^{\circ}}+2.3026\times R\times T\times log\left(Q\right) \end{array}$$

*Q* was calculated according to Equation (9), where *a*_*i*_ is the thermodynamic activity of the *i*^*th*^ chemical species in the overall growth reaction (*r*) and *v*_*i, r*_ is the stoichiometric coefficient for that species.


(8) $$\begin{array}{l} Q=\Pi a_{i}^{v_{i,r}} \end{array}$$

Activities of the aqueous species were calculated using PHREEQC (Parkhurst and Appelo, 2013). Activity of biomass in solution was converted from molality (C-mol/kg solvent) assuming an activity coefficient of 1 (Tebes-Stevens et al., 1998; Xu et al., 1998; Parkhurst and Appelo, 1999; Hunt et al., 2022). The overall change in Gibbs energy during incubation, Δ*G*_*inc*_, for each experiment was calculated with Equation (10) as the difference between the initial Δ*G*_*r*_ available (Δ*G*_*r, initial*_), calculated from the initial chemical composition of the medium solution and biomass, and the final Δ*G*_*r*_ available (Δ*G*_*r, final*_), calculated from the final chemical composition of the medium solution and biomass (see Supplementary material for chemical compositions of the media).


(9) $$\begin{array}{l} \Delta G_{inc}=\Delta G_{r,initial}-\Delta G_{r,final} \end{array}$$

### 2.5. Energy partitioning modeling

Hunt et al. (2022) provides a detailed description of the energy partitioning model used in this work. Here, we present a brief description of this approach. An Arrhenius relationship is applied to the specific energy consumption rate (*q*_*J*_, kJ C-mol^−1^ h^−1^) of a cell that is corrected for temperature (*T*) relative to the reference temperature at 298 K ($q_{J^{{}^{\prime }}}$). This relationship is shown in Equation (11), where *E*_*J*_ is the specific activation energy for energy consumption and *R* is the gas constant. The underlying assumption for this Arrhenius dependence is that a cell obtaining energy is the growth rate limiting step.


(10) $$\begin{array}{l} q_{J}=q_{J^{{}^{\prime }}}\times \exp\left[-\frac{E_{J}}{R}\left(\frac{1}{T}-\frac{1}{298}\right)\right] \end{array}$$

The specific lactate consumption (*q*_*L*_) and specific heat evolution rate (*q*_*H*_) are used as proxies to infer the specific energy consumption rate. These relationships are shown in Equations (11) and (12), where $Y_{\frac{J}{L}}$ is the energy consumed per mole of lactate and $Y_{\frac{H}{J}}$ is the heat evolved per unit of energy consumed.


(11) $$\begin{array}{l} q_{L}=\frac{q_{J^{{}^{\prime }}}}{Y_{\frac{J}{L}}}\times \exp\left[-\frac{E_{J}}{R}\left(\frac{1}{T}-\frac{1}{298}\right)\right] \end{array}$$

(12) $$\begin{array}{l} q_{H}=Y_{\frac{H}{J}}\times q_{J^{{}^{\prime }}}\times \exp\left[-\frac{E_{J}}{R}\left(\frac{1}{T}-\frac{1}{298}\right)\right] \end{array}$$

The heat evolved normalized to C-mol of biomass formed (Δ*H*_*inc*_) is linearly related to the biomass yield (*Y*_*X*/*D*_), $Y_{\frac{J}{L}}$, and $Y_{\frac{H}{J}}$ as shown in Equation (13).


(13) $$\begin{array}{l} \Delta H_{inc}=\frac{Y_{\frac{H}{J}}}{Y_{\frac{L}{J}}}\times \frac{1}{Y_{\frac{X}{L}}} \end{array}$$

The model of Pirt (1982) as amended by Tijhuis et al. (1993) is used to determine the division of the energy consumed into the growth-associated biomass yield [defined as growth-associated costs (GAC)] and costs to maintain viability [defined as maintenance without growth (NGAM)], as shown in Equation (14). Specific energy consumption is the product of the growth rate (μ hour^−1^) and the GAC. NGAM is the temperature-dependent maintenance. Specific lactate consumption rates, specific heat evolution rates, heat evolved per biomass, biomass yield on lactate, and specific growth rate for each experimental replicate were simultaneously fitted to Equations (11)–(14) for parameter estimation. A Jackknife cross-validation approach was used to assess the error of the parameters. This approach estimated parameters from a subset of the data by removing one data point and repeating this process until each data point had been removed. The observations were randomly ordered, and this process was repeated 1,000 times.


(14) $$\begin{array}{l} q_{J}=GAC\times \mu +\left[NGAM\times \exp\left(-\frac{E_{NGAM}}{R}\times \left(\frac{1}{T}-\frac{1}{298}\right)\right)\right] \end{array}$$

## 3. Results

### 3.1. Enthalpies and Gibbs energies of incubation

Microbial growth calorimetry aggregates all biotic and abiotic processes that contribute to Gibbs energy dissipation (Gustafsson, 1991). Systematic bias may be introduced in heat flow measurements for reactions and processes that are not associated with cell growth. Some reactions such as protonation and complexation reactions can be corrected based on the change in the chemical speciation of the media (von Stockar et al., 1993a). We calculated this speciation change in the medium with PHREEQC (Parkhurst and Appelo, 2013) and estimated that the influence of these reactions was less than 8% of the heat evolved in Fe(III)-based incubation and less than 1% of the heat evolved in O_2_-based incubation. Other processes that have the potential to influence the heat flow signal cannot be corrected for, such as cells settling out of solution, cells adhering to the reaction vessel. These processes may introduce additional errors in the calorimetric measurements.

**Figure 1:**
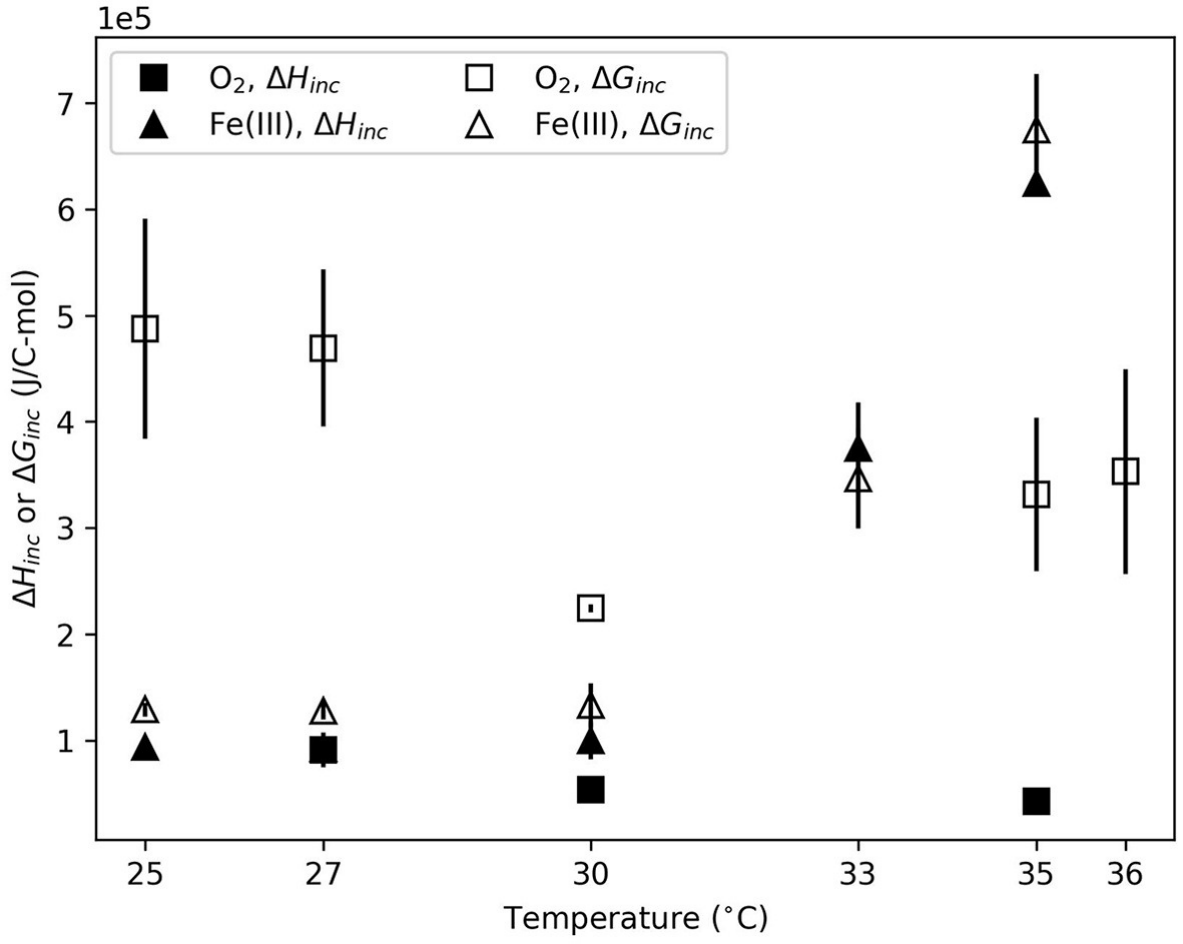
Gibbs energy (Δ*G*_*inc*_) and enthalpy (Δ*H*_*inc*_) of incubation for O_2_- and Fe(III)-based growth as a function of temperature. Error bars reflect standard deviation between replicate experiments.

In the aerobic system, calorimetric data were successfully obtained at 27, 30, and 35°C. At 25 and 36°C, no clear calorimetric signal was consistently observed within 60 h of incubation. For each condition where calorimetric data was obtained, the total heat evolved was normalized to C-mol biomass produced (Figure 1). For aerobic growth, Δ*H*_*inc*_ was significantly more exothermic (*p* = 0.03) at 27°C than at 35°, but not 30°C. Anaerobic growth was also significantly affected by temperature. At 25, 27, and 30°C, Δ*H*_*inc*_ values for anaerobic growth were not different with 95% confidence. Δ*H*_*inc*_ significantly increased (*p* < 0.001) at 33 and 35°C compared to 30°C.

The total amount of Gibbs energy consumed during incubation was also normalized to C-mol biomass produced. Δ*G*_*inc*_, illustrated in Figure 1, shows trends similar to Δ*H*_*inc*_. Δ*G*_*inc*_ for the aerobic data was indistinguishable (*p* > 0.1) at different temperatures. In the anaerobic system, Δ*G*_*inc*_ exhibited an identical statistical trend to Δ*H*_*inc*_. At 25, 27, and 30°C Δ*G*_*inc*_ values were not different (*p* > 0.9). Δ*G*_*inc*_ significantly increased (*p* < 0.005) at 33 and 35°C compared to lower temperatures.

### 3.2. Catabolic cycles

**Figure 2:**
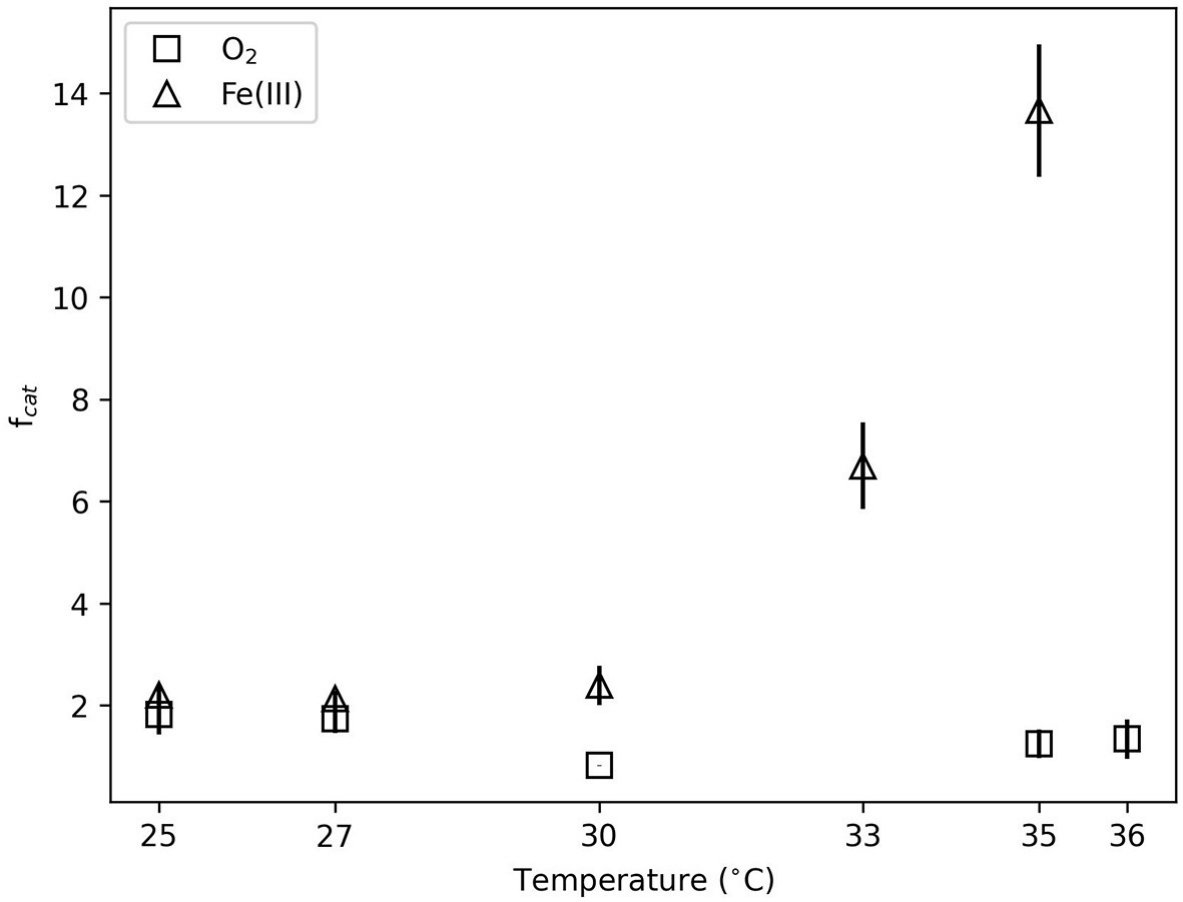
Catabolic cycles to produce observed biomass (*f*_*cat*_, defined in Equation 4) illustrated as a function of temperature. Error bars reflect standard deviation between replicate experiments.

Figure 2 illustrates the *f*_*cat*_ values, which describe the catabolic cycles needed to generate the observed biomass. The anaerobic system had higher *f*_*cat*_ values than the aerobic systems at all temperatures. The aerobic *f*_*cat*_ values ranged from approximately 0.8 to 2 with no significant differences between temperatures (*p* >0.1). Anaerobic *f*_*cat*_ values at 30°C and below were approximately 3 and indistinguishable with temperature (*p* >0.5). Above 30°C, *f*_*cat*_ values increased to approximately 8 and 15 at 33°C and 35°C, respectively. This increase was significant enough to distinguish these *f*_*cat*_ values (*p* < 0.05) from lower temperatures.

### 3.3. Growth stoichiometry

We determined the catabolic reactions for each growth strategy based on Equation (3) and the constraints provided by the changes in the growth medium. As shown in Equations (15) and (16), the stoichiometric ratios of the electron donor and acceptor differed between growth with O_2_ and Fe(III). For aerobic growth, one mole of O_2_ is reduced per mole of oxidized lactate. In Fe(III) growth, 1.33 moles of Fe(III) are reduced per mole of lactate. For both growth systems, lactate was partially oxidized and acetate/acetic acid was produced along with CO_2_. The anabolic reaction, solved according to Equation (2), is the same for aerobic and anaerobic growth, as the electron acceptor is not involved in the reaction, as shown in Equation (17). The reaction involved the conversion of lactate, ammonium, and protons into biomass. The overall growth reactions, as determined with Equations (4) and (5), for aerobic and anaerobic growth are shown in Equations (18) and (19), respectively. Those reactions predict that 0.74 moles of O_2_ will be reduced per mole of lactate in the aerobic system and 0.99 moles of Fe(III) per mole of lactate in the anaerobic system. The observed ratio of O_2_:lactate in the experiments was 1.20 ± 0.23. The Fe(III): lactate ratio in the experiments was 1.46 ± 0.15. These results are more consistent with the stoichiometry for the catabolic reaction than overall growth reaction.


(15) $$\begin{array}{l} C_{3}H_{5}O_{3}^{-}+O_{2}=C_{2}H_{3}O_{2}^{-}+CO_{2}+H_{2}O \end{array}$$

(16) $$\begin{array}{l} C_{3}H_{5}O_{3}^{-}+1.33Fe^{3+}+\;0.33OH^{-}=1.33C_{2}H_{4}O_{2}\;+\;1.33Fe^{2+}+\;0.33CO_{2} \end{array}$$

(17) $$\begin{array}{l} 0.35C_{3}H_{5}O_{3}^{-}+0.2NH_{4}^{+}+0.15H^{+}=CH_{1.8}O_{0.5}N_{0.2}+\\ 0.45H_{2}O+0.05CO_{2} \end{array}$$

(18) $$\begin{array}{c} 1.35C_{3}H_{5}O_{3}^{-}+O_{2}+0.2NH_{4}^{+}+0.15H^{+}=\\ CH_{1.8}O_{0.5}N_{0.2}+C_{2}H_{3}O_{2}^{-}+1.05CO_{2}+1.450H_{2}O \end{array}$$

(19) $$\begin{array}{c} 1.35C_{3}H_{5}O_{3}^{-}\;+\;1.33Fe^{3+}\;+\;0.33OH^{-}\;+\;0.2NH_{4}^{+}+0.15H^{+}=\\ CH_{1.8}O_{0.5}N_{0.2}\;+\;1.33C_{2}H_{4}O_{2}\;+\;1.33Fe^{2+}\;+\;0.38CO_{2}\;+\;0.450H_{2}O \end{array}$$

## 4. Discussion

### 4.1. Temperature impacts

For both growth strategies, an upper limit of the growth temperature was established. Consistent O_2_ growth was not observed above 36°C, and 35°C was the maximum temperature for growth on Fe(III). It is noteworthy that the growth strategies yielded different temperature limits, and this likely reflects differences in the physiological underpinnings of those metabolism. Specifically, the temperature limits for these growth strategies may differ because different enzymes are required for growth on O_2_ and Fe(III) (Myers and Myers, 1993; Blakeney et al., 2000). However, due in large part to the complex nature of electron transfer through the cell envelope and the multiple cytochromes that are thought to be involved in CN32 growth (Blakeney et al., 2000), few details regarding the exact enzymes involved in O_2_ and Fe(III) growth have been quantified. Since the growth temperature limits often depend on the limits of the enzymatic activity (Daniel, 1996), we can infer a similar effect for CN32.

Another factor that may influence the growth temperature limits is the growth medium. Furthermore, since the growth medium was different for aerobic and Fe-based growth (in lactate concentration and Fe(III) vs. O_2_ presence) those differences must be considered when comparing the data. For both media, the ionic strength was approximately 0.04 M. There is some evidence that increasing ionic strength can yield a higher temperature limit for bacterial growth (Nichols et al., 2000). This suggests that if salt concentrations in the medium were increased, the temperature limit could also increase. Fe(III)-based experiments had slightly higher ionic strength (0.045 vs. 0.041 M for the O_2_ experiments) due to the higher concentration of lactate and the ionic nature of the electron acceptor. That difference is much smaller than those that have been observed to incur temperature limit impacts. Therefore, the ionic strength difference between the two grow media was likely not large enough to influence the temperature limits for growth.

The clear relationship between temperature and *f*_*cat*_ for Fe(III)-based growth indicates that the allocation of the energy produced in catabolism also changes with temperature. This is in contrast to aerobic growth, where there was no clear relationship as a function of temperature. Because *f*_*cat*_ reflects the proportion of Gibbs energy used in anabolism, this allocation of energy reflects its relative use in the production of new biomass (anabolism) vs. other non-growth processes. At higher temperatures, the expenditure of energy to maintain sufficient cellular functioning, rather than to reproduce, may explain the statistically significant increase in *f*_*cat*_ above 30°. As has been previously shown, protein instability and aggregation, along with membrane fluidity, increase with temperature, thus affecting cellular homeostasis (de Mendoza and Cronan, 1983; Schramm et al., 2019). These changes require the cell to overcome that physiological stress to maintain its internal functions, which is likely to result in increased energy use.

**Figure 3:**
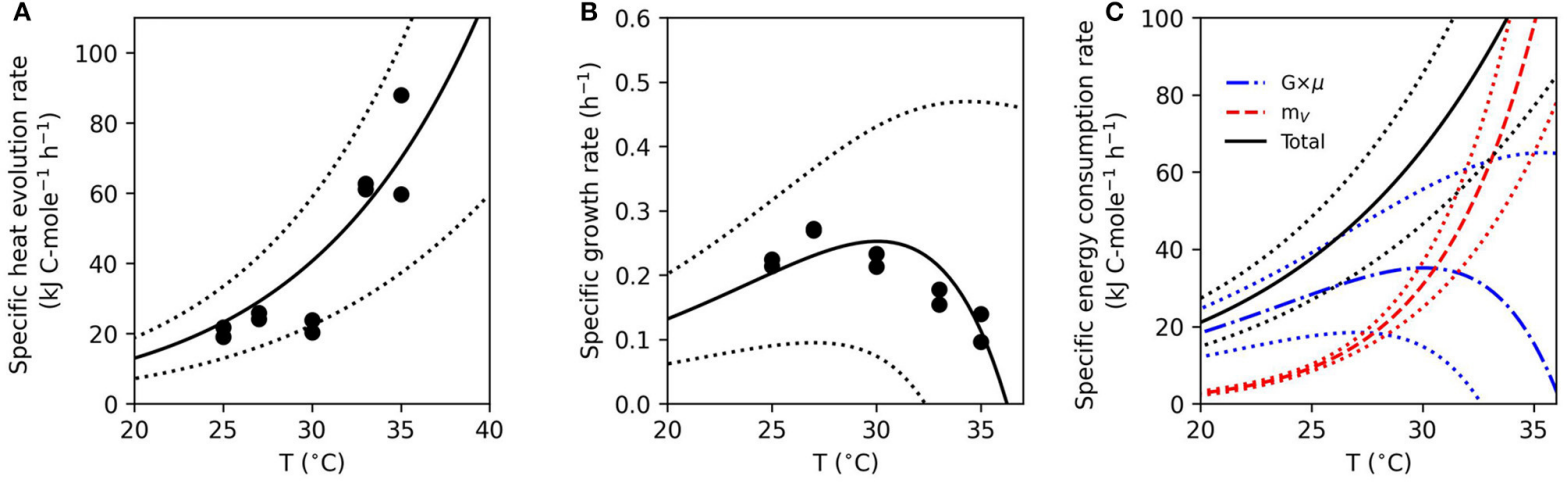
Model fit to measured energy and growth parameters as a function of temperature. Solid curves indicate model predictions of specific heat evolution rate **(A)** and specific growth rate **(B)**. Closed circles are data points for each experimental replicate. Predictions of growth associated costs (*G*×μ) and non-growth associated maintenance (*m*_ν_) are shown by broken curves, while total the specific energy consumption rate is the solid curve **(C)**. The standard deviations of parameters were used to show the error of the model (dotted curves) in all panels.

**Figure 4:**
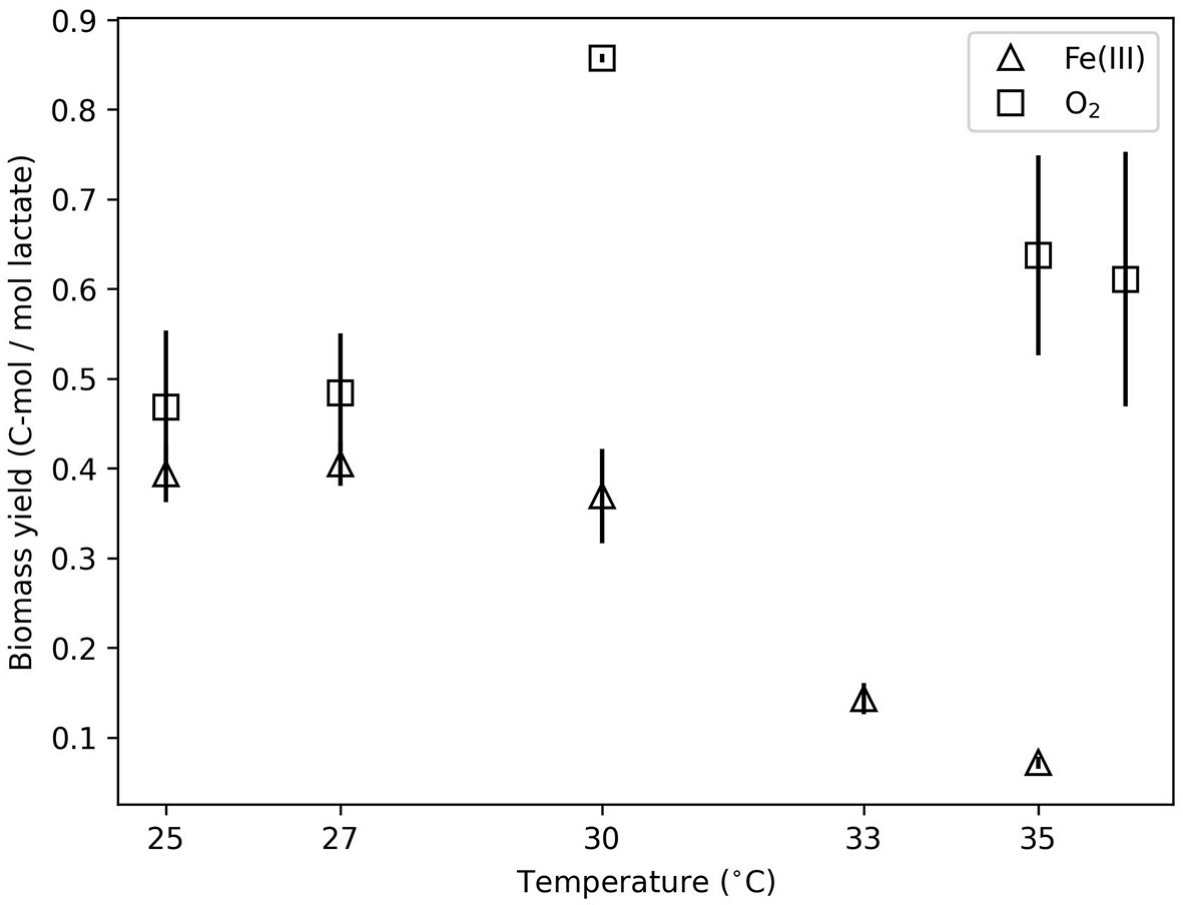
Biomass yield (C-mol/mol lactate) as a function of temperature for O_2_- and Fe(III)-based growth.

The energy partitioning model corroborates the observed increase in *f*_*cat*_ for Fe(III)-based growth. Figure 3A illustrates the fit of the measured specific heat evolution rate to Equation (12). The model predicts an increase in the specific heat evolution rate with temperature, which is consistent with the data. As the temperature increases, the growth rate reaches a maximum at 27°C and then decreases (Figure 3B). The modeling estimates of heat dissipation per Gibbs energy consumed and Gibbs energy consumed per mol lactate from the model fit agreed with the thermodynamics-based calculations (Supplementary Figures 5, 6). Figure 3C illustrates the predicted partition of the specific energy consumption rate into GAC (*G*×μ) and NGAM (*m*_*V*_). Below 30°C, more than 50% of the specific energy consumption rate is predicted to be divided into GAC. This is consistent with the observed biomass yield, which is shown as a function of temperature in Figure 4. The biomass yield above 30°C showed a distinct decrease compared to lower temperatures. Data suggest that between 30 and 33°C is a tipping point where temperature stress diverts a substantial amount of energy away from GAC to NGAM. The energy partitioning model predicts this “tipping point” temperature to be ≈31°C where GAC is equal to NGAM.

It is important to note that the modeling in this work based biomass yield on cell counts. Energetic resources diverted to the production of organic carbon exudates were not measurable given the small sample volumes (3 ml), multiple analyses performed (cell counts, substrate consumption, and metabolite production), the dilution needed for the measurement, and the presence of organic carbon from the electron donor and waste products. Consequently, if exudate or biofilm production is not measured and taken into account when calculating the total C-mol biomass produced, the biomass yield and *f*_*cat*_ values will be impacted. Macroscopic instances of biofilm were not observed, however, biofilm is typically observed by scanning electron microscopy (Alhede et al., 2012). Smaller biofilms may have been missed with the fluorescence microscopy used in this study. Two of the variables that influence exudate/biofilm production were included in these experiments: O_2_ and elevated temperatures.

For *Shewanella* strains in particular, the presence of O_2_ has been shown to dramatically increase exudate/biofilm production (Wu et al., 2013). In contrast, Yan et al. (2020) and Pinel et al. (2021) have shown that elevated temperature reduces exudate/biofilm formation in other taxa. In Fe(III) growth experiments, we expect a lesser, if any, amount of exudate/biofilm production. In aerobic experiments, these impacts could be substantial. The aerobic data showed only a slight influence of the temperature. Biomass yield at 30°C was significantly (*p* < 0.05) higher than at 25°C, and Δ*H*_*inc*_ at 27°C was more exothermic than higher temperatures (see above). The lack of a clear influence of temperature and lack of calorimetric data at temperature extremes precludes energy partitioning modeling and any maintenance energy insights for aerobic growth. The lack of consistent temperature influence may be due to undetectable exudate/biomass that would alter biomass yield. This impact would be expected to occur more frequently at lower temperatures, where oxygen solubility is also higher. Consequently, aerobic incubation may be affected by temperature more strongly than was detected in this work.

### 4.2. Geochemical implications

Considering growth efficiency across conditions can be helpful in understanding the physiological limits of microbial life and the potential for geochemical impacts of metabolism. Microbial growth efficiency is often interpreted from parameters such as biomass yield (del Giorgio and Cole, 1998). However, from a thermodynamic perspective, efficiency is an output/input ratio in energy units (Gnaiger, 1990). This can be applied to microbial growth when energy consumption is modeled in terms of catabolic and anabolic reactions (e.g., Calabrese et al., 2021). The ratio of Δ*G*_*inc*_ used by the anabolic reaction ($\Delta G_{inc}^{An}$) relative to the Gibbs energy released by the catabolic reaction ($\Delta G_{inc}^{Cat}$) scaled with *f*_*cat*_ is used here to consider the thermodynamic efficiency of microbial growth (η, Equation 20) under experimental conditions.


(20) $$\begin{array}{l} \eta =\frac{\Delta G_{inc}^{An}}{f_{cat}\times \Delta G_{inc}^{Cat}} \end{array}$$

**Figure 5:**
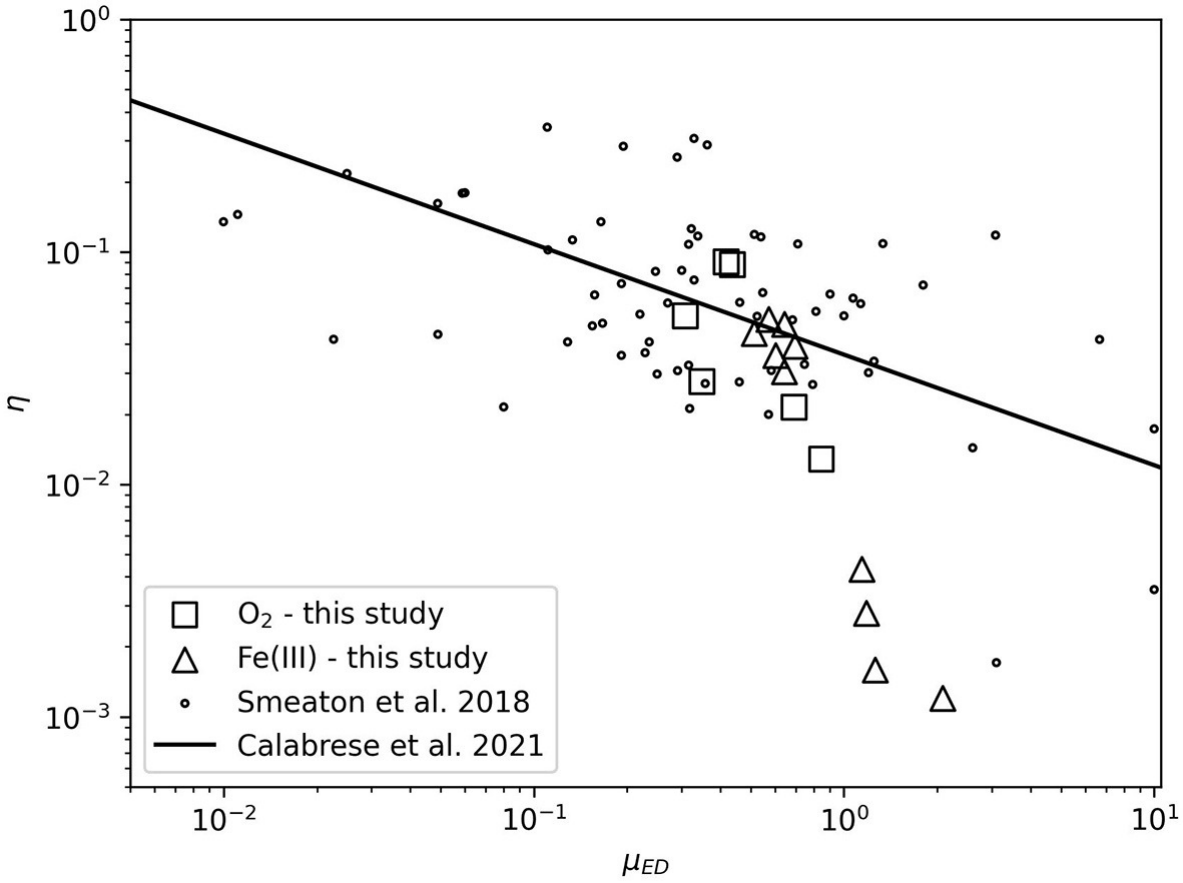
Thermodynamic efficiency (η) as a function of electron donor uptake rate (μ_*ED*_). O_2_- and Fe(III)-based growth data are plotted along with compilation data from Smeaton and Van Cappellen (2018) and the power law scaling relationship between η and μ_*ED*_ from Calabrese et al. (2021).

Figure 5 illustrates the thermodynamic efficiency of growth experiments in this study relative to the compilation of Smeaton and Van Cappellen (2018). Calabrese et al. (2021) identified a power-law scaling relationship between the thermodynamic efficiency of microbial growth and the electron donor uptake rate (μ_*ED*_). A major geochemical implication of this relationship is that estimations of thermodynamic efficiency can be made from commonly measured parameters in microbial growth investigations (specific growth rate and biomass yield). This provides the means to improve biogeochemical modeling.

The data collected in this study that do not exhibit significant temperature stress generally agree with the power-law scaling relationship of Calabrese et al. (2021). Aerobic data generally cluster with the majority of data. The anaerobic data at 30°C and below cluster tightly along the scaling relationship. Fitting a new power-law scaling relationship to the data of Smeaton and Van Cappellen (2018) including the aerobic data and anaerobic data at 30°C and below from this work produces slope and intercept values of −0.506 ± 0.202 and −1.469 ± 0.118, respectively. These parameters are statistically indistinguishable from the slope [−0.476 with 2σ confidence interval (−0.719, −0.271)], and intercept [−1.443 with 2σ confidence interval (−1.579, −1.306)] values from Calabrese et al. (2021). Experiments with thermodynamic efficiencies less than 10^−2^ are poorly predicted by the power-law scaling relationship. This result is not surprising in the context of the various lines of evidence showing the impact of temperature stress on iron incubations at 33 and 35°C: Δ*H*_*inc*_ and Δ*G*_*inc*_ increase, catabolic cycles increase, NGAM increases, and biomass yield decreases. The thermodynamic efficiency of microbial growth may generally deviate from this power-law scaling relationship when under extreme stress, such as nearing the maximum growth temperature. This is an area for future work to better understand potential geochemical impacts when catabolic cycles are high and biomass production is limited.

Making biogeochemical predictions based on thermodynamic modeling of microbial growth is predicated on the ability to describe growth with chemical reactions that reasonably reflect chemical consumption and/or metabolite production. For example, Equation (21) illustrates the established stoichiometry for Fe(III) growth by *S. putrefaciens* as described in Lovley (1993). This stoichiometry has been applied without verification (Tran et al., 2020).


(21) $$\begin{array}{l} C_{3}H_{5}O_{3}^{-}\;+\;4Fe^{3+}\;+\;2H_{2}O=C_{2}H_{3}O_{2}\;+\;HCO_{3}^{-}\;+\;4Fe^{2+}\;+\;5H^{+} \end{array}$$

The measurements made in this work, however, are consistent with a very different reaction stoichiometry (see Equation 16). Geochemical predictions of Fe(II) production based on Equation (21) would over-predict production approximately 3 times according to the present work. These differences are substantial and could alter geochemical predictions of mineral phase stability, redox conditions, ionic strength, and similar variables. Consequently, verification of reasonable chemical equations of microbial growth is critical to accurate geochemical modeling.

**Figure 6:**
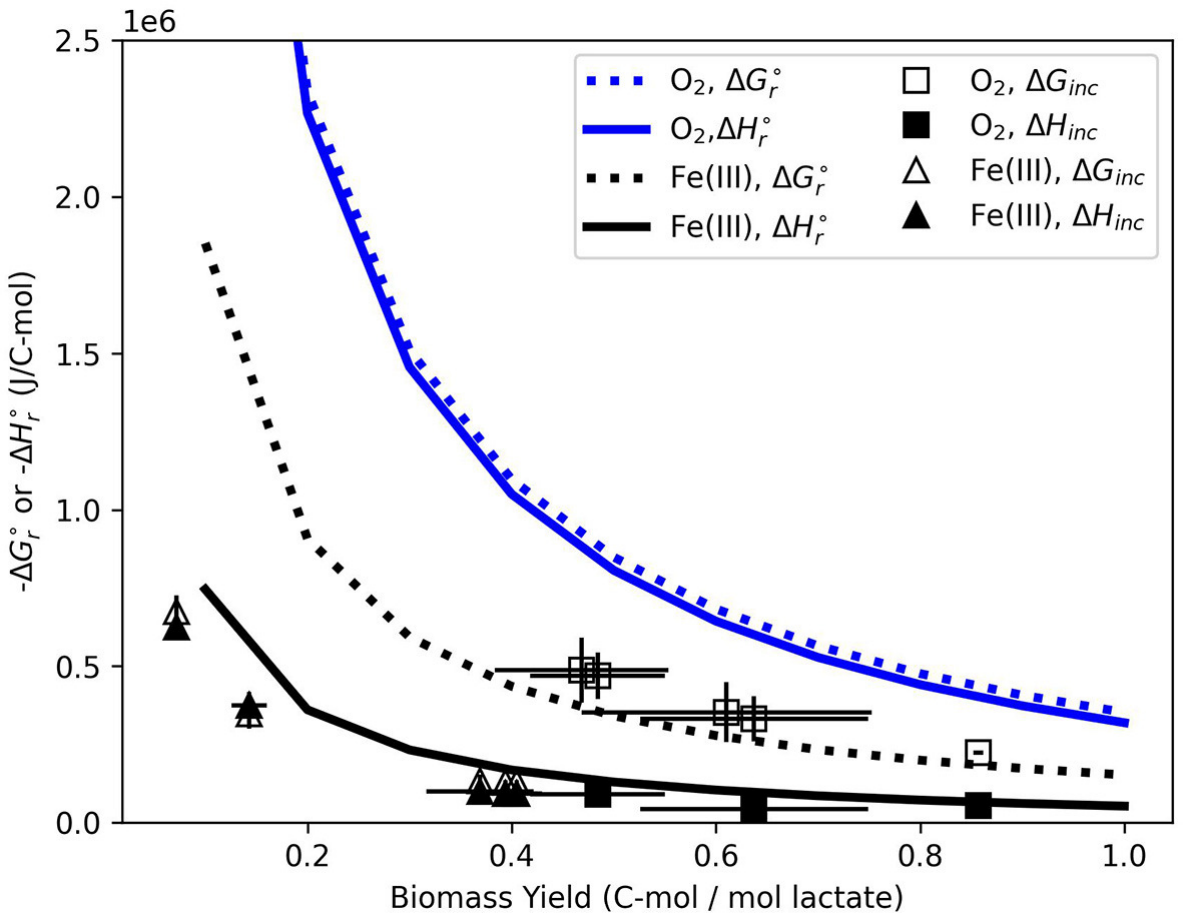
Curves represent standard state values for $\Delta G_{r}^{{}^{\circ}}$ and $\Delta H_{r}^{{}^{\circ}}$ for both growth strategies. The curve thickness spans the variation in thermodynamic potentials with temperature from 25 to 36°C. Δ*G*_*inc*_ and Δ*H*_*inc*_ for O_2_- and Fe(III)-based growth are plotted at as data points. Error bars reflect the standard deviation between replicate samples.

Another approach to predicting microbial biomass yield or chemical changes in the absence of microbial growth data is based solely on standard state parameters that describe an overall growth equation. Standard state values for both O_2_ and Fe(III)-based growth, for 1 C-mol of biomass, are illustrated in Figure 6 together with measured values of Δ*G*_*inc*_ and Δ*H*_*inc*_. The thickness of curves spans the variation in standard state predictions from 25 to 36°C. For O_2_ growth, $\Delta G_{r}^{{}^{\circ}}$ is slightly more negative than $\Delta H_{r}^{{}^{\circ}}$. This predicts that the entropic component of growth ($\Delta S_{r}^{{}^{\circ}}$) is minimal, which agrees with the data (Δ*G*_*inc*_≈Δ*H*_*inc*_). At the highest biomass yield (≈0.84 C-mol/mol lactate at 30°C), the aerobic data are the closest to predictions, which are ≈6 times more exergonic/exothermic. Standard state predictions of growth on Fe(III) show the same general trend as O_2_ ($\Delta G_{r}^{{}^{\circ}}$ more negative than $\Delta H_{r}^{{}^{\circ}}$), but the magnitude of that difference is much greater. Consequently, the entropic component of growth is predicted to be more significant. This can be conceptualized by considering the metabolite stream during growth. Production of small metabolites would increase entropy during growth, thus helping drive $\Delta G_{r}^{{}^{\circ}}$ negative (von Stockar and Liu, 1999). The data do not show a significant entropic component during incubation; that is, Δ*H*_*inc*_ is very similar to Δ*G*_*inc*_. Similarly to the aerobic system, Fe(III) growth is closest to standard state predictions at the highest biomass yields (≈0.33 C-mol/mol lactate at ≥30°C). This suggests that when *f*_*cat*_ is minimized or η is maximized, we observe experimental data that come closest to standard state predictions. Consequently, predictions based on standard state parameters may be most useful under ideal conditions for growth when maximum coupling between the catabolic and anabolic cycles are achieved.

## 5. Conclusions

In this work, we present the first quantitative comparison of aerobic and anaerobic growth by *Shewanella putrefaciens* CN32. Through a combination of calorimetric analysis and measured changes in a defined growth medium, we describe the bioenergetics of these growth strategies. These data demonstrated a minimal effect of temperature on aerobic growth, while temperature substantially impacted anaerobic growth. Energy partitioning modeling provided context for the observed temperature stress during growth on Fe(III) and predicted a tipping point temperature where growth should substantially decrease. Ultimately, the ability to incorporate microbial growth reactions into geochemical modeling requires predictive strategies that provide reasonable predictions. Stoichiometries of growth reactions that reflect substrate consumption and the metabolite product are an essential component of those predictions. We found that the growth of CN32 on Fe(III) was more reasonably described with a different stoichiometry than has previously been applied. This further motivates the continued investigation of microbial bioenergetics, as the use of theoretical growth stoichiometries may lead to inaccurate predictions regarding the effects of growth. We evaluated two geochemical approaches to predict CN32 growth based on thermodynamic microbial growth efficiency and standard state thermodynamic potentials of the overall growth reactions. Generally, predictions based on thermodynamic microbial growth efficiency were more reasonable than standard state predictions, and predictions may be most reasonable under ideal growth conditions. In all, this work provides data crucial to accurately describing the growth of a common iron-reducing subsurface bacterium under a range of conditions and advances our understanding of its role in both oxygenated and anaerobic systems.

## Data availability statement

The original contributions presented in the study are included in the article/Supplementary material, further inquiries can be directed to the corresponding author.

## Author contributions

AW and DG-L contributed to conception and design of the study, analyzed the data, and wrote sections of the manuscript. Both authors read and approved the submitted version.
