# Supplementary material for: Bioenergetics of aerobic and anaerobic growth of Shewanella putrefaciens CN32
Source: Front Microbiol. 2023 Aug 2;14:1234598. doi: 10.3389/fmicb.2023.1234598 (PMC10433392; doi:10.3389/fmicb.2023.1234598)
Supplement: Supplementary file 1 [file Data_Sheet_1.PDF]

Supplementary Material

1 ADDITIONAL EXPERIMENTAL DETAILS

Table S1. Defined medium for *Shewanella putrefaciens* CN32 adjusted to ≈ pH 7.8 for growth on iron and ≈ pH 8 for aerobic growth.

Compound	mM	mL/L
sodium DL-lactate	10[1]	
HEPES	10	
sodium bicarbonate	2	
magnesium sulfate heptahydrate	1	
ammonium sulfate	0.095	
boric acid	0.057	
monobasic potassium phosphate	0.05	
dibasic potassium phosphate	0.05	
Wolfe's Mineral Solution	–	5
Wolfe's Vitamin Solution	–	10
L-arginine HCl	0.095	
L-glutamine	0.11	
DL-serine	0.38	

[1] 15mm for growth on iron

Table S2. Wolfe's Mineral Solution

Compound	Amount	Unit	Amount [g/L]	Conc. [mM]
Nitrilotriacetic acid	1.5	g	1.5	7.848
MgSO4	3	g	3	24.923
MnSO4	0.5	g	0.5	3.311
NaCl	1	g	1	17.112
FeSO4	0.1	g	0.1	0.658
CaCl2	0.1	g	0.1	0.901
CoCl2	0.1	g	0.1	0.770
ZnSO4	0.1	g	0.1	0.620
CuSO4	0.01	g	0.01	0.063
AlK(SO4)2	0.01	g	0.01	0.039
H3BO3	0.01	g	0.01	0.162
Na2MoO4	0.01	g	0.01	0.049
Distilled water	1000	ml		

Table S3. Wolfe's Vitamin Solution

Compound	Amount	Unit	Amount [g/L]	Conc. [mM]
Biotin	2	mg	0.002	0.008
Folic acid	2	mg	0.002	0.005
Pyridoxine hydrochloride	10	mg	0.01	0.049
Thiamine-HCl x 2 H2O	5	mg	0.005	0.013
Riboflavin	5	mg	0.005	0.013
Nicotinic acid	5	mg	0.005	0.041
D-Calcium pantothenate	5	mg	0.005	0.021
Vitamin B12	0.1	mg	0.0001	0.000
p-Aminobenzoic acid	5	mg	0.005	0.036
Lipoic acid	5	mg	0.005	0.024
Distilled water	1000	ml		

2 SUPPLEMENTARY FIGURES

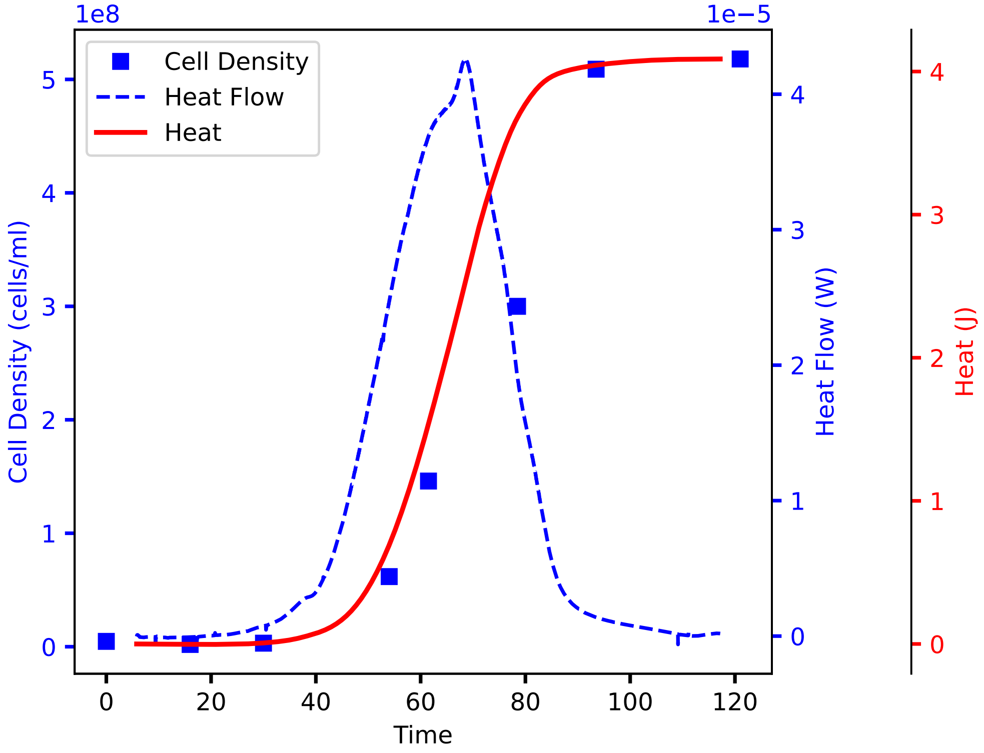

Figure S1: Calorimetric and corresponding cell density data of *S. putrefaciens* CN32 growth on iron with 120 mM lactate. This is a higher substrate concentration than used in experimental conditions; however, it clearly shows the correlation between cumulative heat and growth phase. Heat plateaus in stationary phase. Destructive sampling of parallel cultures were used to determine cell densities.

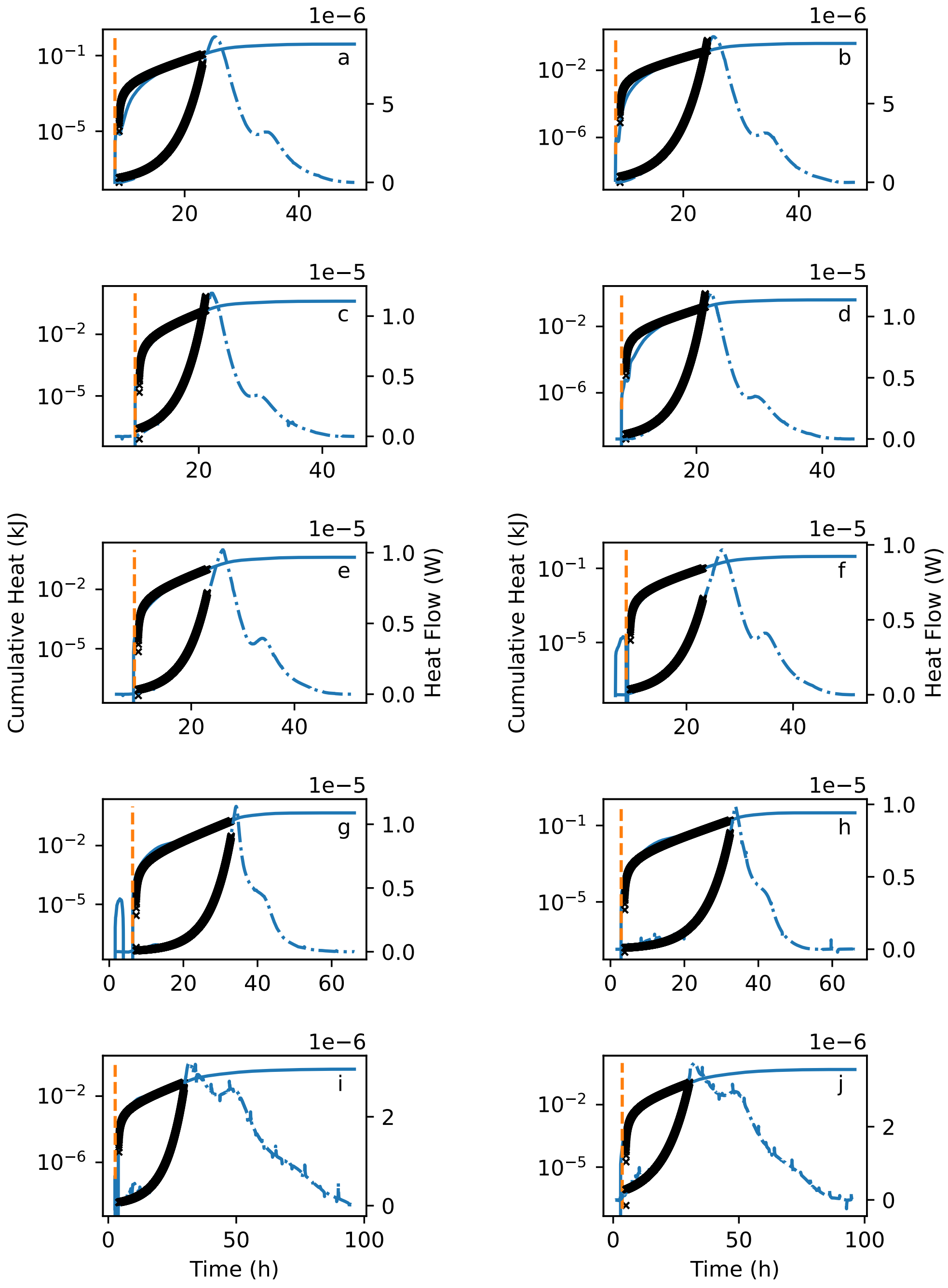

Figure S2: Fit of an exponential model to the calorimetric data of *S. putrefaciens* CN32 growth on iron at 25°C (a, b), 27°C (c, d), 30°C (e, f), 33°C (g, h), and 35°C (i, j). Lag phase is indicated by the broken vertical line. The solid curve and broken curves illustrate the cumulative heat and heat flow, respectively. The 'X' markers (appears as thick black curve) indicate the fit of the exponential model.

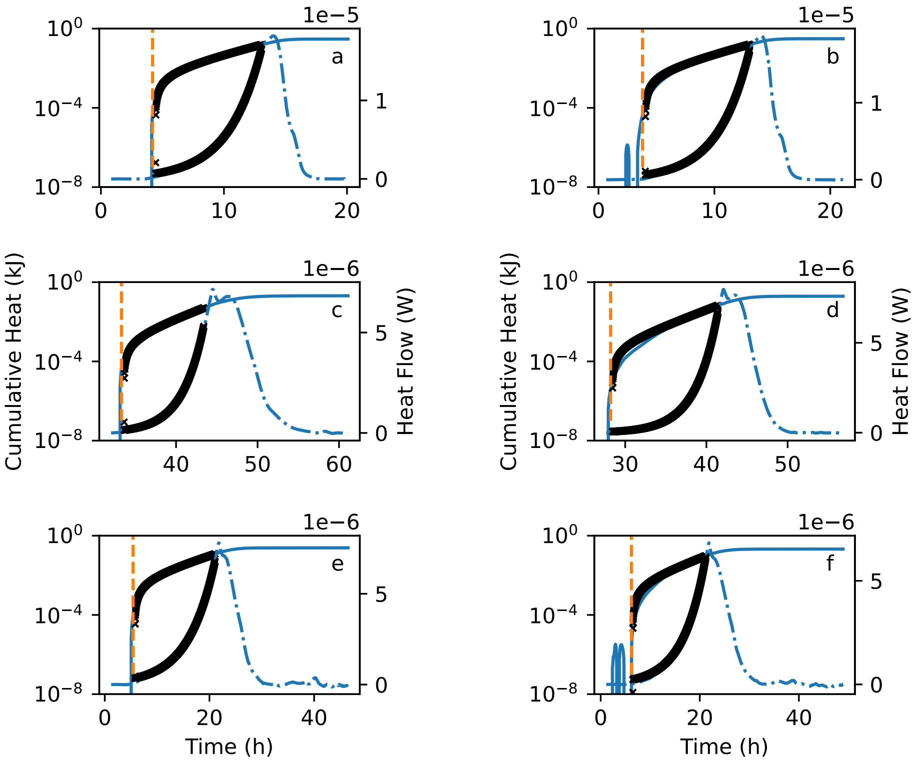

Figure S3: Fit of an exponential model to the calorimetric data of *S. putrefaciens* CN32 growth on oxygen at 27°C (a, b), 30°C (c, d), and 35°C (e, f). Lag phase is indicated by the broken vertical line. The solid curve and broken curves illustrate the cumulative heat and heat flow, respectively. The 'X' markers (appears as thick black curve) indicate the fit of the exponential model.

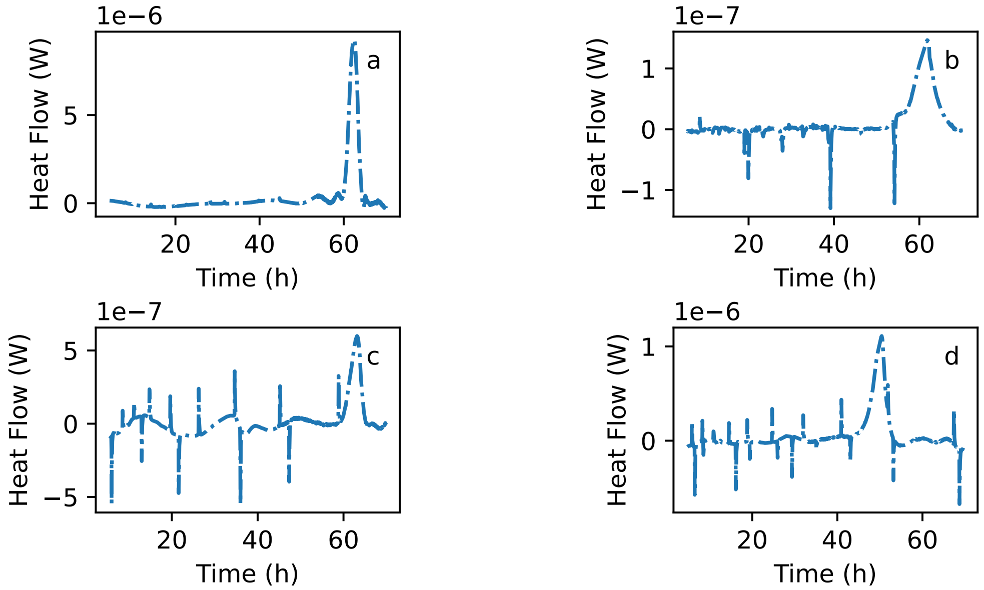

Figure S4: Calorimetric data of *S. putrefaciens* CN32 growth on oxygen at 25°C (a, b) and 36°C. The broken curves illustrate the heat flow. Data was inconsistent between replicates; therefore, the data was not included in the analysis.

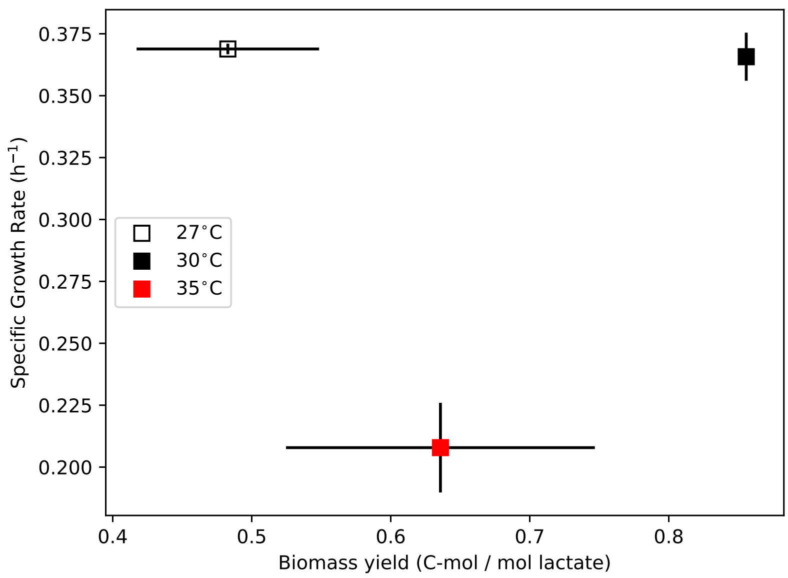

Figure S5: Specific growth rates as a function of biomass yield for aerobic growth. Error bars reflect standard deviation between replicate experiments.

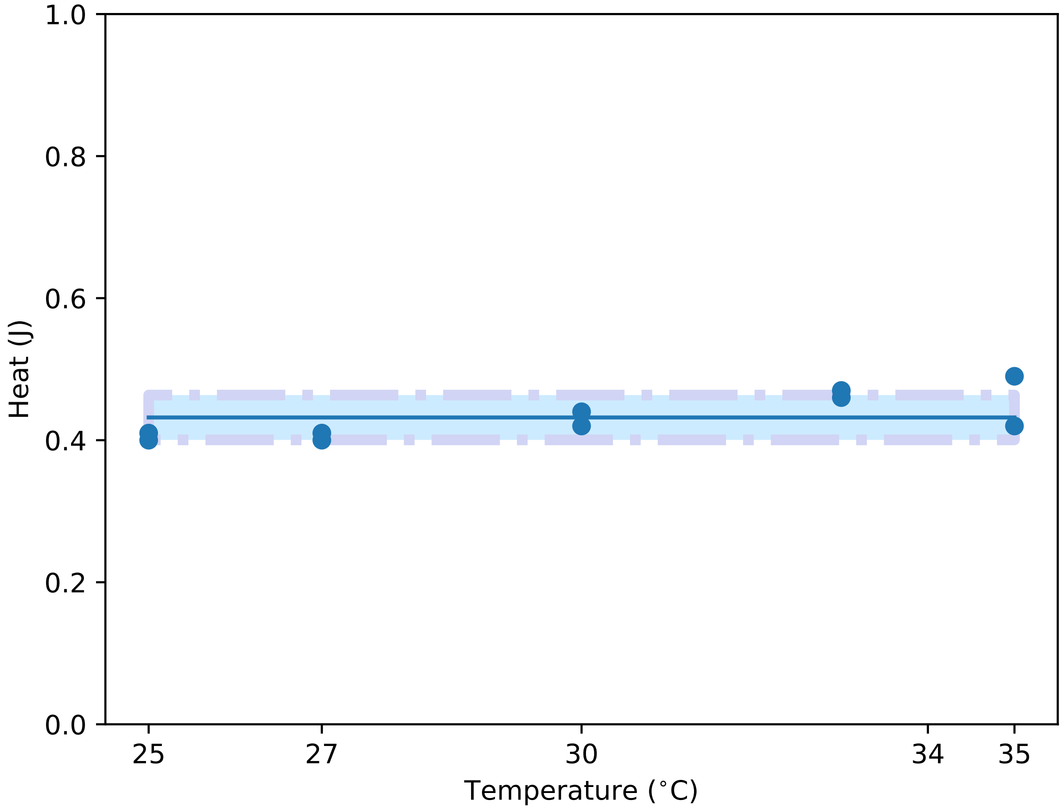

Figure S6: Heat evolved during growth on iron as a function of temperature. Solid line represents the average heat evolved, and the shaded region is 1σ.

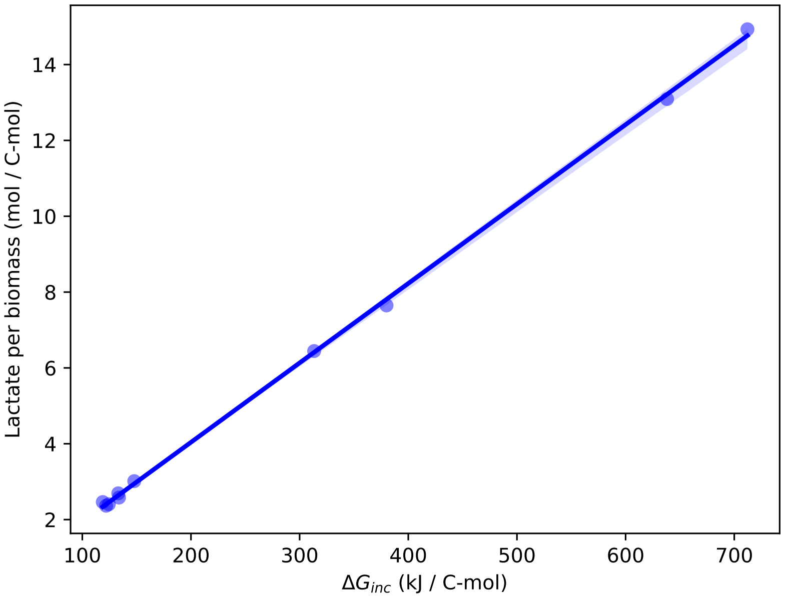

Figure S7: Lactate consumed per C-mol of biomass produced as a function of ΔG_{inc}. $Y_{\frac{J}{L}}$ is the reciprocal of the slope of the linear regression shown by the solid curve. The shaded region illustrates the 95% confidence interval of the regression. $Y_{\frac{J}{L}}$ determined from ΔG_{inc} is 48.5 ± 0.6. $Y_{\frac{J}{L}}$ determined from the energy partitioning modeling is 41.6 ± 14.4, which is within error of the parameter determined from the thermodynamic data.

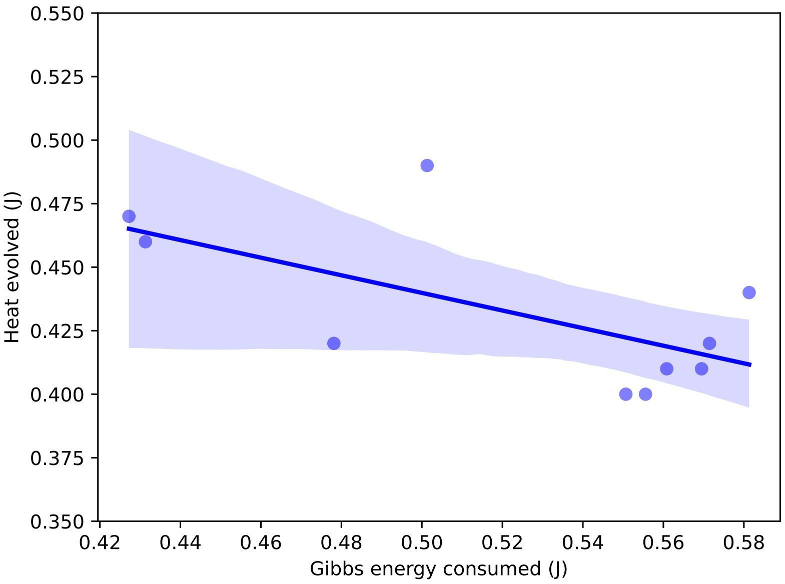

Figure S8: Heat evolved as a function of Gibbs energy consumed during incubation. $Y_{\frac{H}{J}}$ is of the slope of the linear regression shown by the solid curve, which represents the heat evolved per Gibbs energy consumed. The shaded region illustrates the 95% confidence interval of the regression. $Y_{\frac{H}{J}}$ determined from the linear regression is 0.81 ± 0.09. $Y_{\frac{H}{J}}$ determined from the energy partitioning modeling is 0.62 ± 0.21, which is within error of the parameter determined from the thermodynamic data.

Table S4. Initial chemical composition for iron incubation. Element / ion concentrations in mmol/kgw. Temperature in °C.

	FeCal25-ch1	FeCal25-ch2	FeCal27-ch1	FeCal27-ch2	FeCal30-ch1	FeCal30-ch2	FeCal33-ch1	FeCal33-ch2	FeCal35-ch1	FeCal35-ch2
Temp	2.500E+01	2.500E+01	2.700E+01	2.700E+01	3.000E+01	3.000E+01	3.300E+01	3.300E+01	3.500E+01	3.500E+01
pH	7.819E+00	7.819E+00	7.691E+00	7.691E+00	7.756E+00	7.756E+00	7.686E+00	7.686E+00	7.702E+00	7.702E+00
Acetate	8.595E-02	8.595E-02	8.595E-02	8.595E-02	8.595E-02	8.595E-02	8.595E-02	8.595E-02	8.595E-02	8.595E-02
Al	1.936E-04	1.936E-04	1.936E-04	1.936E-04	1.936E-04	1.936E-04	1.936E-04	1.936E-04	1.936E-04	1.936E-04
B	5.781E-02	5.781E-02	5.781E-02	5.781E-02	5.781E-02	5.781E-02	5.781E-02	5.781E-02	5.781E-02	5.781E-02
C(4)	2.000E+00	2.000E+00	2.000E+00	2.000E+00	2.000E+00	2.000E+00	2.000E+00	2.000E+00	2.000E+00	2.000E+00
Ca	4.505E-03	4.505E-03	4.505E-03	4.505E-03	4.505E-03	4.505E-03	4.505E-03	4.505E-03	4.505E-03	4.505E-03
Citrate	6.791E+00	6.791E+00	6.791E+00	6.791E+00	6.791E+00	6.791E+00	6.791E+00	6.791E+00	6.791E+00	6.791E+00
Cl	1.973E-01	1.973E-01	1.973E-01	1.973E-01	1.973E-01	1.973E-01	1.973E-01	1.973E-01	1.973E-01	1.973E-01
Co	3.851E-03	3.851E-03	3.851E-03	3.851E-03	3.851E-03	3.851E-03	3.851E-03	3.851E-03	3.851E-03	3.851E-03
Cu(2)	3.133E-04	3.133E-04	3.133E-04	3.133E-04	3.133E-04	3.133E-04	3.133E-04	3.133E-04	3.133E-04	3.133E-04
Fe(2)	1.810E-01	1.810E-01	1.360E-01	1.360E-01	1.810E-01	1.810E-01	1.360E-01	1.360E-01	2.020E-01	2.020E-01
Fe(3)	6.791E+00	6.791E+00	6.791E+00	6.791E+00	6.791E+00	6.791E+00	6.791E+00	6.791E+00	6.791E+00	6.791E+00
Hepes	1.000E+01	1.000E+01	1.000E+01	1.000E+01	1.000E+01	1.000E+01	1.000E+01	1.000E+01	1.000E+01	1.000E+01
K	1.502E-01	1.502E-01	1.502E-01	1.502E-01	1.502E-01	1.502E-01	1.502E-01	1.502E-01	1.502E-01	1.502E-01
Lactate	1.719E+01	1.719E+01	1.719E+01	1.719E+01	1.719E+01	1.719E+01	1.719E+01	1.719E+01	1.719E+01	1.719E+01
Mg	1.125E+00	1.125E+00	1.125E+00	1.125E+00	1.125E+00	1.125E+00	1.125E+00	1.125E+00	1.125E+00	1.125E+00
Mn(2)	1.656E-02	1.656E-02	1.656E-02	1.656E-02	1.656E-02	1.656E-02	1.656E-02	1.656E-02	1.656E-02	1.656E-02
Mo	2.428E-04	2.428E-04	2.428E-04	2.428E-04	2.428E-04	2.428E-04	2.428E-04	2.428E-04	2.428E-04	2.428E-04
N(-3)	1.900E-01	1.900E-01	1.900E-01	1.900E-01	1.900E-01	1.900E-01	1.900E-01	1.900E-01	1.900E-01	1.900E-01
Na	2.657E+01	2.657E+01	2.657E+01	2.657E+01	2.657E+01	2.657E+01	2.657E+01	2.657E+01	2.657E+01	2.657E+01
P	1.000E-01	1.000E-01	1.000E-01	1.000E-01	1.000E-01	1.000E-01	1.000E-01	1.000E-01	1.000E-01	1.000E-01
S(6)	1.243E+00	1.243E+00	1.243E+00	1.243E+00	1.243E+00	1.243E+00	1.243E+00	1.243E+00	1.243E+00	1.243E+00
Zn	3.098E-03	3.098E-03	3.098E-03	3.098E-03	3.098E-03	3.098E-03	3.098E-03	3.098E-03	3.098E-03	3.098E-03

Table S5. Final chemical composition for iron incubation. Element / ion concentrations in mmol/kgw. Temperature in °C.

	FeCal25-ch1	FeCal25-ch2	FeCal27-ch1	FeCal27-ch2	FeCal30-ch1	FeCal30-ch2	FeCal33-ch1	FeCal33-ch2	FeCal35-ch1	FeCal35-ch2
Temp	2.500E+01	2.500E+01	2.700E+01	2.700E+01	3.000E+01	3.000E+01	3.300E+01	3.300E+01	3.500E+01	3.500E+01
pH	8.355E+00	8.356E+00	8.292E+00	8.294E+00	8.288E+00	8.294E+00	8.221E+00	8.221E+00	8.220E+00	8.233E+00
Acetate	3.620E+00	5.090E+00	7.160E+00	4.850E+00	2.840E+00	3.210E+00	5.660E+00	6.070E+00	3.920E+00	6.060E+00
Al	1.936E-04	1.936E-04	1.936E-04	1.936E-04	1.936E-04	1.936E-04	1.936E-04	1.936E-04	1.936E-04	1.936E-04
B	5.781E-02	5.781E-02	5.781E-02	5.781E-02	5.781E-02	5.781E-02	5.781E-02	5.781E-02	5.781E-02	5.781E-02
C(4)	2.000E+00	2.000E+00	2.000E+00	2.000E+00	2.000E+00	2.000E+00	2.000E+00	2.000E+00	2.000E+00	2.000E+00
Ca	4.505E-03	4.505E-03	4.505E-03	4.505E-03	4.505E-03	4.505E-03	4.505E-03	4.505E-03	4.505E-03	4.505E-03
Citrate	6.791E+00	6.791E+00	6.791E+00	6.791E+00	6.791E+00	6.791E+00	6.791E+00	6.791E+00	6.791E+00	6.791E+00
Cl	1.973E-01	1.973E-01	1.973E-01	1.973E-01	1.973E-01	1.973E-01	1.973E-01	1.973E-01	1.973E-01	1.973E-01
Co	3.851E-03	3.851E-03	3.851E-03	3.851E-03	3.851E-03	3.851E-03	3.851E-03	3.851E-03	3.851E-03	3.851E-03
Cu(2)	3.133E-04	3.133E-04	3.133E-04	3.133E-04	3.133E-04	3.133E-04	3.133E-04	3.133E-04	3.133E-04	3.133E-04
Fe(2)	6.694E+00	6.793E+00	6.334E+00	6.550E+00	6.736E+00	6.701E+00	5.886E+00	5.984E+00	6.393E+00	6.206E+00
Fe(3)	1.300E-01	8.000E-02	9.000E-02	8.000E-02	1.800E-01	1.700E-01	1.900E-01	1.700E-01	3.200E-01	3.100E-01
Hepes	1.000E+01	1.000E+01	1.000E+01	1.000E+01	1.000E+01	1.000E+01	1.000E+01	1.000E+01	1.000E+01	1.000E+01
K	1.502E-01	1.502E-01	1.502E-01	1.502E-01	1.502E-01	1.502E-01	1.502E-01	1.502E-01	1.502E-01	1.502E-01
Lactate	1.220E+01	1.246E+01	1.245E+01	1.255E+01	1.201E+01	1.201E+01	1.335E+01	1.339E+01	1.278E+01	1.270E+01
Mg	1.125E+00	1.125E+00	1.125E+00	1.125E+00	1.125E+00	1.125E+00	1.125E+00	1.125E+00	1.125E+00	1.125E+00
Mn(2)	1.656E-02	1.656E-02	1.656E-02	1.656E-02	1.656E-02	1.656E-02	1.656E-02	1.656E-02	1.656E-02	1.656E-02
Mo	2.428E-04	2.428E-04	2.428E-04	2.428E-04	2.428E-04	2.428E-04	2.428E-04	2.428E-04	2.428E-04	2.428E-04
N(-3)	1.900E-01	1.900E-01	1.900E-01	1.900E-01	1.900E-01	1.900E-01	1.900E-01	1.900E-01	1.900E-01	1.900E-01
Na	2.657E+01	2.657E+01	2.657E+01	2.657E+01	2.657E+01	2.657E+01	2.657E+01	2.657E+01	2.657E+01	2.657E+01
P	1.000E-01	1.000E-01	1.000E-01	1.000E-01	1.000E-01	1.000E-01	1.000E-01	1.000E-01	1.000E-01	1.000E-01
S(6)	1.243E+00	1.243E+00	1.243E+00	1.243E+00	1.243E+00	1.243E+00	1.243E+00	1.243E+00	1.243E+00	1.243E+00
Zn	3.098E-03	3.098E-03	3.098E-03	3.098E-03	3.098E-03	3.098E-03	3.098E-03	3.098E-03	3.098E-03	3.098E-03

Table S6. Auxilliary data for iron incubation. Q = heat evolved. mu = specific growth rate. muED = electron donor uptake rate. Yx/L = biomass yield.

	FeCal25-ch1	FeCal25-ch2	FeCal27-ch1	FeCal27-ch2	FeCal30-ch1	FeCal30-ch2	FeCal33-ch1	FeCal33-ch2	FeCal35-ch1	FeCal35-ch2
T (C)	2.500e+01	2.500e+01	2.700e+01	2.700e+01	3.000e+01	3.000e+01	3.300e+01	3.300e+01	3.500e+01	3.500e+01
Q (J)	4.100e-01	4.000e-01	4.100e-01	4.000e-01	4.200e-01	4.400e-01	4.700e-01	4.600e-01	4.200e-01	4.900e-01
mu (1/h)	2.240e-01	2.139e-01	2.695e-01	2.723e-01	2.326e-01	2.134e-01	1.774e-01	1.546e-01	1.396e-01	9.644e-02
muED (mol lac / h)	6.844e-01	5.872e-01	7.805e-01	7.274e-01	6.445e-01	7.229e-01	1.310e+00	1.360e+00	2.372e+00	1.427e+00
initial cell counts (cells/ml)	1.350e+06	1.350e+06	1.170e+06	1.170e+06	9.810e+05	9.810e+05	1.200e+06	1.200e+06	1.120e+05	1.120e+05
final cell counts (cells/ml)	6.780e+07	7.180e+07	6.830e+07	7.240e+07	7.680e+07	6.300e+07	2.270e+07	1.910e+07	1.070e+07	1.250e+07
Yx/L (C-mol / mol lac)	3.273e-01	3.643e-01	3.453e-01	3.743e-01	3.609e-01	2.952e-01	1.354e-01	1.137e-01	5.884e-02	6.757e-02
Biomass (C-mol)	4.214e-06	4.468e-06	4.257e-06	4.517e-06	4.808e-06	3.933e-06	1.363e-06	1.135e-06	6.714e-07	7.856e-07

Table S7. Activities for iron incubation.

	FeCal25-ch1	FeCal25-ch2	FeCal27-ch1	FeCal27-ch2	FeCal30-ch1	FeCal30-ch2	FeCal33-ch1	FeCal33-ch2	FeCal35-ch1	FeCal35-ch2
aNH4+ initial	1.473e-04	1.473e-04	1.480e-04	1.480e-04	1.474e-04	1.474e-04	1.477e-04	1.477e-04	1.474e-04	1.474e-04
aH+ initial	1.517e-08	1.517e-08	2.037e-08	2.037e-08	1.754e-08	1.754e-08	2.061e-08	2.061e-08	1.986e-08	1.986e-08
aC3H5O3- initial	1.378e-02	1.378e-02	1.383e-02	1.383e-02	1.378e-02	1.378e-02	1.380e-02	1.380e-02	1.378e-02	1.378e-02
aCH3COOH initial	5.920e-08	5.920e-08	7.971e-08	7.971e-08	6.841e-08	6.841e-08	8.045e-08	8.045e-08	7.745e-08	7.745e-08
aH2O initial	9.988e-01	9.988e-01	9.988e-01	9.988e-01	9.988e-01	9.988e-01	9.988e-01	9.988e-01	9.988e-01	9.988e-01
aCO2 initial	5.267e-05	5.267e-05	7.138e-05	7.138e-05	6.257e-05	6.257e-05	7.428e-05	7.428e-05	7.209e-05	7.209e-05
aFe+2 initial	4.052e-07	4.052e-07	3.399e-07	3.399e-07	4.301e-07	4.301e-07	3.465e-07	3.465e-07	5.131e-07	5.131e-07
aFe+3 initial	2.455e-13	2.455e-13	4.753e-13	4.753e-13	3.451e-13	3.451e-13	4.875e-13	4.875e-13	4.539e-13	4.539e-13
aNH4+ final	1.518e-04	1.516e-04	1.511e-04	1.515e-04	1.517e-04	1.517e-04	1.506e-04	1.506e-04	1.513e-04	1.508e-04
aH+ final	4.416e-09	4.406e-09	5.105e-09	5.082e-09	5.152e-09	5.082e-09	6.012e-09	6.012e-09	6.026e-09	5.848e-09
aC3H5O3- final	1.000e-02	1.020e-02	1.017e-02	1.027e-02	9.838e-03	9.833e-03	1.087e-02	1.091e-02	1.044e-02	1.035e-02
aCH3COOH final	7.360e-07	1.031e-06	1.680e-06	1.134e-06	6.730e-07	7.502e-07	1.562e-06	1.675e-06	1.085e-06	1.627e-06
aH2O final	9.989e-01	9.989e-01	9.988e-01	9.989e-01	9.989e-01	9.989e-01	9.989e-01	9.988e-01	9.989e-01	9.989e-01
aCO2 final	1.561e-05	1.556e-05	1.827e-05	1.821e-05	1.877e-05	1.851e-05	2.213e-05	2.213e-05	2.223e-05	2.153e-05
aFe+2 final	1.603e-04	1.793e-04	1.043e-04	1.344e-04	1.685e-04	1.613e-04	6.639e-05	7.293e-05	1.123e-04	9.120e-05
aFe+3 final	1.069e-16	6.531e-17	1.230e-16	1.076e-16	2.541e-16	2.291e-16	4.539e-16	4.055e-16	7.709e-16	6.745e-16

Table S8. Thermodynamic data for iron incubation. i = initial. f = final. dG = Gibbs energy. fcat = catabolic cycles. Cat = catabolic. Ana = anabolic. dHinc = Enthalpy of incubation. eta = theromdynamic efficiency.

	FeCal25-ch1	FeCal25-ch2	FeCal27-ch1	FeCal27-ch2	FeCal30-ch1	FeCal30-ch2	FeCal33-ch1	FeCal33-ch2	FeCal35-ch1	FeCal35-ch2
iAffinity (J/C-mol)	4.571e+05	4.038e+05	4.419e+05	4.015e+05	4.206e+05	5.237e+05	1.179e+06	1.407e+06	2.784e+06	2.436e+06
fAffinity (J/C-mol)	3.240e+05	2.794e+05	3.081e+05	2.796e+05	3.017e+05	3.758e+05	8.657e+05	1.027e+06	2.072e+06	1.798e+06
dG C-mol (J/C-mol)	1.331e+05	1.244e+05	1.338e+05	1.219e+05	1.189e+05	1.478e+05	3.133e+05	3.800e+05	7.121e+05	6.381e+05
dG (J)	5.608e-01	5.556e-01	5.695e-01	5.507e-01	5.715e-01	5.813e-01	4.272e-01	4.313e-01	4.781e-01	5.013e-01
fcat	2.343e+00	2.054e+00	2.231e+00	2.016e+00	2.113e+00	2.664e+00	6.095e+00	7.298e+00	1.458e+01	1.274e+01
Cat iAffinity (J/C-mol)	4.304e+05	3.770e+05	4.145e+05	3.742e+05	3.925e+05	4.956e+05	1.151e+06	1.379e+06	2.750e+06	2.402e+06
Cat fAffinity (J/cmol)	3.075e+05	2.631e+05	2.915e+05	2.631e+05	2.852e+05	3.589e+05	8.458e+05	1.007e+06	2.050e+06	1.777e+06
Cat dG (J)	5.175e-01	5.091e-01	5.237e-01	5.015e-01	5.158e-01	5.377e-01	4.163e-01	4.227e-01	4.699e-01	4.914e-01
Ana dG (J)	4.330e-02	4.647e-02	4.576e-02	4.918e-02	5.567e-02	4.359e-02	1.091e-02	8.577e-03	8.205e-03	9.925e-03
dHinc (kJ/C-mol)	9.730e+04	8.953e+04	9.631e+04	8.855e+04	8.735e+04	1.119e+05	3.447e+05	4.052e+05	6.255e+05	6.237e+05
eta	3.571e-02	4.443e-02	3.916e-02	4.865e-02	5.109e-02	3.043e-02	4.298e-03	2.780e-03	1.197e-03	1.585e-03

Table S9. Initial chemical composition for aerobic incubation. Element / ion concentrations in mmol/kgw. Temperature in °C.

	OxCal25-ch1	OxCal25-ch2	OxCal27-ch1	OxCal27-ch2	OxCal30-ch1	OxCal30-ch2	OxCal35-ch1	OxCal35-ch2	OxCal36-ch1	OxCal36-ch2
Temp	2.500E+01	2.500E+01	2.700E+01	2.700E+01	3.000E+01	3.000E+01	3.500E+01	3.500E+01	3.600E+01	3.600E+01
pH	8.006E+00	8.006E+00	8.178E+00	8.178E+00	8.097E+00	8.097E+00	8.036E+00	8.036E+00	8.145E+00	8.145E+00
Acetate	2.000E-01	2.000E-01	2.000E-01	2.000E-01	2.000E-01	2.000E-01	2.000E-01	2.000E-01	2.000E-01	2.000E-01
Al	1.936E-04	1.936E-04	1.936E-04	1.936E-04	1.936E-04	1.936E-04	1.936E-04	1.936E-04	1.936E-04	1.936E-04
B	5.781E-02	5.781E-02	5.781E-02	5.781E-02	5.781E-02	5.781E-02	5.781E-02	5.781E-02	5.781E-02	5.781E-02
C(4)	2.000E+00	2.000E+00	2.000E+00	2.000E+00	2.000E+00	2.000E+00	2.000E+00	2.000E+00	2.000E+00	2.000E+00
Ca	4.505E-03	4.505E-03	4.505E-03	4.505E-03	4.505E-03	4.505E-03	4.505E-03	4.505E-03	4.505E-03	4.505E-03
Cl	1.973E-01	1.973E-01	1.973E-01	1.973E-01	1.973E-01	1.973E-01	1.973E-01	1.973E-01	1.973E-01	1.973E-01
Co	3.851E-03	3.851E-03	3.851E-03	3.851E-03	3.851E-03	3.851E-03	3.851E-03	3.851E-03	3.851E-03	3.851E-03
Cu(2)	3.133E-04	3.133E-04	3.133E-04	3.133E-04	3.133E-04	3.133E-04	3.133E-04	3.133E-04	3.133E-04	3.133E-04
Fe(2)	1.798E-03	1.798E-03	1.798E-03	1.798E-03	1.798E-03	1.798E-03	1.798E-03	1.798E-03	1.798E-03	1.798E-03
Hepes	1.000E+01	1.000E+01	1.000E+01	1.000E+01	1.000E+01	1.000E+01	1.000E+01	1.000E+01	1.000E+01	1.000E+01
K	1.502E-01	1.502E-01	1.502E-01	1.502E-01	1.502E-01	1.502E-01	1.502E-01	1.502E-01	1.502E-01	1.502E-01
Lactate	1.088E+01	1.088E+01	1.139E+01	1.139E+01	1.053E+01	1.053E+01	1.053E+01	1.053E+01	1.031E+01	1.031E+01
Mg	1.125E+00	1.125E+00	1.125E+00	1.125E+00	1.125E+00	1.125E+00	1.125E+00	1.125E+00	1.125E+00	1.125E+00
Mn(2)	1.656E-02	1.656E-02	1.656E-02	1.656E-02	1.656E-02	1.656E-02	1.656E-02	1.656E-02	1.656E-02	1.656E-02
Mo	2.428E-04	2.428E-04	2.428E-04	2.428E-04	2.428E-04	2.428E-04	2.428E-04	2.428E-04	2.428E-04	2.428E-04
N(-3)	1.900E-01	1.900E-01	1.900E-01	1.900E-01	1.900E-01	1.900E-01	1.900E-01	1.900E-01	1.900E-01	1.900E-01
Na	2.657E+01	2.657E+01	2.657E+01	2.657E+01	2.657E+01	2.657E+01	2.657E+01	2.657E+01	2.657E+01	2.657E+01
P	1.000E-01	1.000E-01	1.000E-01	1.000E-01	1.000E-01	1.000E-01	1.000E-01	1.000E-01	1.000E-01	1.000E-01
S(6)	1.243E+00	1.243E+00	1.243E+00	1.243E+00	1.243E+00	1.243E+00	1.243E+00	1.243E+00	1.243E+00	1.243E+00
Zn	3.098E-03	3.098E-03	3.098E-03	3.098E-03	3.098E-03	3.098E-03	3.098E-03	3.098E-03	3.098E-03	3.098E-03

Table S10. Final chemical composition for aerobic incubation. Element / ion concentrations in mmol/kgw. Temperature in °C.

	OxCal25-ch1	OxCal25-ch2	OxCal27-ch1	OxCal27-ch2	OxCal30-ch1	OxCal30-ch2	OxCal35-ch1	OxCal35-ch2	OxCal36-ch1	OxCal36-ch2
Temp	2.500E+01	2.500E+01	2.700E+01	2.700E+01	3.000E+01	3.000E+01	3.500E+01	3.500E+01	3.600E+01	3.600E+01
pH	7.668E+00	7.645E+00	7.649E+00	7.659E+00	7.628E+00	7.625E+00	7.598E+00	7.576E+00	7.639E+00	7.664E+00
Acetate	9.940E-01	1.126E+00	3.009E+00	2.772E+00	1.060E+00	2.970E-01	2.850E+00	2.242E+00	6.720E-01	1.809E+00
Al	1.936E-04	1.936E-04	1.936E-04	1.936E-04	1.936E-04	1.936E-04	1.936E-04	1.936E-04	1.936E-04	1.936E-04
B	5.781E-02	5.781E-02	5.781E-02	5.781E-02	5.781E-02	5.781E-02	5.781E-02	5.781E-02	5.781E-02	5.781E-02
C(4)	2.000E+00	2.000E+00	2.000E+00	2.000E+00	2.000E+00	2.000E+00	2.000E+00	2.000E+00	2.000E+00	2.000E+00
Ca	4.505E-03	4.505E-03	4.505E-03	4.505E-03	4.505E-03	4.505E-03	4.505E-03	4.505E-03	4.505E-03	4.505E-03
Cl	1.973E-01	1.973E-01	1.973E-01	1.973E-01	1.973E-01	1.973E-01	1.973E-01	1.973E-01	1.973E-01	1.973E-01
Co	3.851E-03	3.851E-03	3.851E-03	3.851E-03	3.851E-03	3.851E-03	3.851E-03	3.851E-03	3.851E-03	3.851E-03
Cu(2)	3.133E-04	3.133E-04	3.133E-04	3.133E-04	3.133E-04	3.133E-04	3.133E-04	3.133E-04	3.133E-04	3.133E-04
Fe(2)	1.798E-03	1.798E-03	1.798E-03	1.798E-03	1.798E-03	1.798E-03	1.798E-03	1.798E-03	1.798E-03	1.798E-03
Hepes	1.000E+01	1.000E+01	1.000E+01	1.000E+01	1.000E+01	1.000E+01	1.000E+01	1.000E+01	1.000E+01	1.000E+01
K	1.502E-01	1.502E-01	1.502E-01	1.502E-01	1.502E-01	1.502E-01	1.502E-01	1.502E-01	1.502E-01	1.502E-01
Lactate	8.005E+00	8.819E+00	8.019E+00	7.846E+00	7.205E+00	7.374E+00	7.088E+00	7.680E+00	7.413E+00	7.966E+00
Mg	1.125E+00	1.125E+00	1.125E+00	1.125E+00	1.125E+00	1.125E+00	1.125E+00	1.125E+00	1.125E+00	1.125E+00
Mn(2)	1.656E-02	1.656E-02	1.656E-02	1.656E-02	1.656E-02	1.656E-02	1.656E-02	1.656E-02	1.656E-02	1.656E-02
Mo	2.428E-04	2.428E-04	2.428E-04	2.428E-04	2.428E-04	2.428E-04	2.428E-04	2.428E-04	2.428E-04	2.428E-04
N(-3)	1.900E-01	1.900E-01	1.900E-01	1.900E-01	1.900E-01	1.900E-01	1.900E-01	1.900E-01	1.900E-01	1.900E-01
Na	2.657E+01	2.657E+01	2.657E+01	2.657E+01	2.657E+01	2.657E+01	2.657E+01	2.657E+01	2.657E+01	2.657E+01
P	1.000E-01	1.000E-01	1.000E-01	1.000E-01	1.000E-01	1.000E-01	1.000E-01	1.000E-01	1.000E-01	1.000E-01
S(6)	1.243E+00	1.243E+00	1.243E+00	1.243E+00	1.243E+00	1.243E+00	1.243E+00	1.243E+00	1.243E+00	1.243E+00
Zn	3.098E-03	3.098E-03	3.098E-03	3.098E-03	3.098E-03	3.098E-03	3.098E-03	3.098E-03	3.098E-03	3.098E-03

Table S11. Auxilliary data for aerobic incubation. Q = heat evolved. mu = specific growth rate. Yx/L = biomass yield.

	OxCal25-ch1	OxCal25-ch2	OxCal27-ch1	OxCal27-ch2	OxCal30-ch1	OxCal30-ch2	OxCal35-ch1	OxCal35-ch2	OxCal36-ch1	OxCal36-ch2
T (C)	2.500e+01	2.500e+01	2.700e+01	2.700e+01	3.000e+01	3.000e+01	3.500e+01	3.500e+01	3.600e+01	3.600e+01
Q (J)			3.704e-01	3.661e-01	3.696e-01	3.603e-01	1.950e-01	2.219e-01		
mu (1/h)			3.704e-01	3.674e-01	3.726e-01	3.589e-01	1.950e-01	2.207e-01		
initial cell counts (cells/ml)	1.150e+06	1.150e+06	1.470e+06	1.470e+06	1.040e+06	1.040e+06	1.020e+06	1.020e+06	6.900e+05	6.900e+05
final cell counts (cells/ml)	1.830e+08	1.690e+08	2.300e+08	2.930e+08	4.400e+08	4.200e+08	2.990e+08	3.160e+08	2.280e+08	2.570e+08
Yx/L (C-mol / mol lac)	4.075e-01	5.285e-01	4.364e-01	5.292e-01	8.523e-01	8.592e-01	5.572e-01	7.143e-01	5.089e-01	7.088e-01
Biomass (C-mol)	2.883e-06	2.661e-06	3.623e-06	4.622e-06	6.959e-06	6.642e-06	4.724e-06	4.994e-06	3.604e-06	4.063e-06

Table S12. Activities for aerobic incubation.

	OxCal25-ch1	OxCal25-ch2	OxCal27-ch1	OxCal27-ch2	OxCal30-ch1	OxCal30-ch2	OxCal35-ch1	OxCal35-ch2	OxCal36-ch1	OxCal36-ch2
aNH4+ initial	1.546e-04	1.546e-04	1.541e-04	1.541e-04	1.542e-04	1.542e-04	1.540e-04	1.540e-04	1.539e-04	1.539e-04
aH+ initial	9.863e-09	9.863e-09	6.637e-09	6.637e-09	7.998e-09	7.998e-09	9.204e-09	9.204e-09	7.161e-09	7.161e-09
aC3H5O3- initial	9.039e-03	9.039e-03	9.443e-03	9.443e-03	8.732e-03	8.732e-03	8.720e-03	8.720e-03	8.531e-03	8.531e-03
aCH3COO- initial	1.625e-04	1.625e-04	1.622e-04	1.622e-04	1.622e-04	1.622e-04	1.620e-04	1.620e-04	1.619e-04	1.619e-04
aO2 initial	2.276e-04	2.276e-04	2.297e-04	2.297e-04	2.329e-04	2.329e-04	2.381e-04	2.381e-04	2.391e-04	2.391e-04
aH2O initial	9.991e-01	9.991e-01	9.991e-01	9.991e-01	9.991e-01	9.991e-01	9.991e-01	9.991e-01	9.991e-01	9.991e-01
aCO2 initial	3.520e-05	3.520e-05	2.401e-05	2.401e-05	2.939e-05	2.939e-05	3.440e-05	3.440e-05	2.684e-05	2.684e-05
aNH4+ final	1.556e-04	1.555e-04	1.552e-04	1.552e-04	1.556e-04	1.557e-04	1.551e-04	1.552e-04	1.552e-04	1.549e-04
aH+ final	2.148e-08	2.265e-08	2.244e-08	2.193e-08	2.355e-08	2.371e-08	2.523e-08	2.655e-08	2.296e-08	2.168e-08
aC3H5O3- final	6.685e-03	7.360e-03	6.683e-03	6.540e-03	6.015e-03	6.159e-03	5.901e-03	6.396e-03	6.176e-03	6.625e-03
aCH3COO- final	8.110e-04	9.181e-04	2.451e-03	2.258e-03	8.646e-04	2.423e-04	2.319e-03	1.824e-03	5.470e-04	1.470e-03
aO2 final	5.585e-49	7.112e-49	2.618e-48	2.355e-48	2.099e-47	2.123e-47	6.622e-46	8.054e-46	7.852e-46	6.324e-46
aH2O final	9.991e-01	9.991e-01	9.991e-01	9.991e-01	9.992e-01	9.992e-01	9.991e-01	9.991e-01	9.992e-01	9.991e-01
aCO2 final	7.638e-05	8.035e-05	8.061e-05	7.885e-05	8.600e-05	8.662e-05	9.361e-05	9.827e-05	8.576e-05	8.100e-05

Table S13. Thermodynamic data for aerobic incubation. i = initial. f = final. dG = Gibbs energy. fcat = catabolic cycles. Cat = catabolic. Ana = anabolic. dHinc = Enthalpy of incubation. eta = theromdynamic efficiency.

	OxCal25-ch1	OxCal25-ch2	OxCal27-ch1	OxCal27-ch2	OxCal30-ch1	OxCal30-ch2	OxCal35-ch1	OxCal35-ch2	OxCal36-ch1	OxCal36-ch2
iAffinity (J/C-mol)	1.100e+06	8.157e+05	1.018e+06	8.130e+05	4.487e+05	4.439e+05	7.659e+05	5.642e+05	8.552e+05	5.715e+05
fAffinity (J/C-mol)	5.395e+05	4.013e+05	4.968e+05	3.962e+05	2.219e+05	2.223e+05	3.834e+05	2.836e+05	4.339e+05	2.867e+05
dG C-mol (J/C-mol)	5.608e+05	4.144e+05	5.215e+05	4.168e+05	2.268e+05	2.216e+05	3.825e+05	2.806e+05	4.213e+05	2.848e+05
dG (J)	1.617e+00	1.103e+00	1.890e+00	1.926e+00	1.579e+00	1.472e+00	1.807e+00	1.401e+00	1.518e+00	1.157e+00
fcat	2.099e+00	1.542e+00	1.935e+00	1.534e+00	8.214e-01	8.121e-01	1.440e+00	1.046e+00	1.611e+00	1.057e+00
Cat iAffinity (J/C-mol)	5.270e+05	5.188e+05	5.269e+05	5.206e+05	5.090e+05	5.089e+05	5.185e+05	5.125e+05	5.221e+05	5.134e+05
Cat fAffinity (J/cmol)	-2.124e+04	1.168e+05	1.799e+04	1.170e+05	2.975e+05	3.024e+05	1.506e+05	2.466e+05	1.157e+05	2.437e+05
Cat dG (J)	1.580e+00	1.070e+00	1.844e+00	1.865e+00	1.472e+00	1.372e+00	1.738e+00	1.328e+00	1.465e+00	1.096e+00
Ana dG (J)	3.640e-02	3.282e-02	4.564e-02	6.113e-02	1.064e-01	1.006e-01	6.914e-02	7.336e-02	5.365e-02	6.156e-02
dHinc (kJ/C-mol)			1.022e+05	7.921e+04	5.312e+04	5.424e+04	4.129e+04	4.443e+04		
eta	1.097e-02	1.989e-02	1.279e-02	2.136e-02	8.798e-02	9.032e-02	2.762e-02	5.281e-02	2.274e-02	5.315e-02
